# Synthesis and structural characterization of the *Z*-isomer of 1,2-bis(tetrazol-5-yl)ethylene (H_2_bte) *via* photoinduced isomerization

**DOI:** 10.1039/d5ra09255h

**Published:** 2026-01-13

**Authors:** Junhao Shi, Sicheng Liao, Shiliang Huang, Tianyu Jiang, Kangcai Wang, Wenquan Zhang

**Affiliations:** a Institute of Chemical Materials, China Academy of Engineering Physics (CAEP) Mianyang Sichuan 621000 China zhangwq-cn@caep.cn

## Abstract

This study demonstrates the photoinduced *E*→*Z* isomerization of 1,2-bis(tetrazol-5-yl)ethylene (H_2_bte), leading to the first successful growth and structural characterization of its *Z*-isomer single crystal. Through optimized 365 nm UV irradiation and subsequent recrystallization, phase-pure *Z*-1,2-bis(tetrazol-5-yl)ethylene (*Z*-H_2_bte) (91.9%) was obtained. Single-crystal X-ray diffraction analysis uncovered a non-planar molecular structure for *Z*-H_2_bte, which fosters a distinct hydrogen-bonding network and crystal packing architecture compared to the planar *E*-isomer. The two isomers were further applied to the construction of sodium and cesium-based metal complexes, with the findings indicating that both isomers present a similar packing and connection mode. This work establishes an experimental foundation for understanding the *E*/*Z* photoisomerization and concomitant structural evolution in the H_2_bte system, while providing a potential pathway for modulating energetic molecular properties through photoinduced *cis*–*trans* isomerization.

## Abstract 2

*Z*-1,2-Bis(tetrazol-5-yl)ethylene was synthesized, crystals were obtained and the photochemical and physicochemical properties revealed. Sodium and cesium complexes of the *E*/*Z*-isomer were constructed and their coordination modes were elucidated.

## Graphical abstract

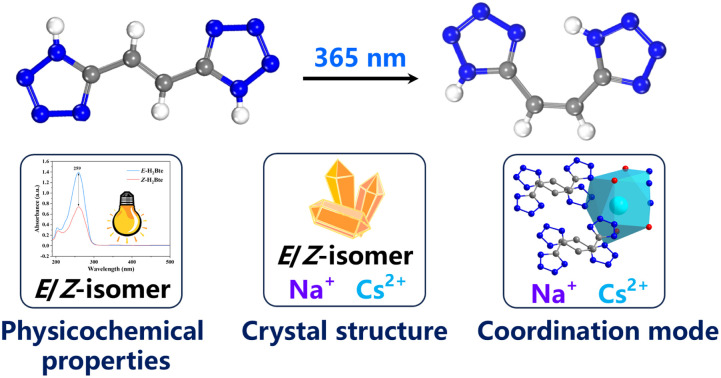

## Introduction

**Fig. 1:**
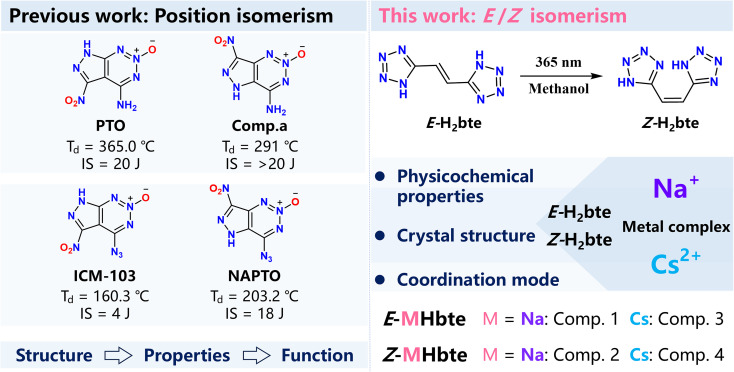
Previous works explored positional isomerism of energetic molecules, whereas this work focuses on *E*/*Z* isomerism. ICM-103: 6-nitro-7-azido-pyrazolo[3,4-*d*][1,2,3]triazine-2-oxide, NAPTO: 4-nitro-7-azido-pyrazol-[3,4-*d*]-1,2,3-triazine-2-oxide.

The design of energetic materials is fundamentally guided by molecular architecture, wherein isomerism constitutes a structurally distinctive element.^1–7^ As depicted in Fig. 1, comparative analyses have established that positional isomerism exerts a pronounced influence on molecular stability.^8,9^ This is exemplified by representative compounds such as 4-amino-5-nitro-7*H*-pyrazolo[3,4-*d*][1,2,3]triazine-2-oxide (PTO) and 4-amino-7-nitro-5*H*-pyrazolo[4,3-*d*][1,2,3]triazine-2-oxide (Comp. a): although possessing identical molecular formulas, positional isomers frequently display discernible variations across multiple structural dimensions, including molecular geometry, electron density profiles, modes of intermolecular interaction, and crystalline packing arrangements.^10–14^ These structural distinctions are intimately linked to essential material attributes such as lattice stabilization energy, inherent bond strength, and energy release dynamics.^15^

Recent years have witnessed substantial expansion in photochemical reaction studies. This rapidly evolving field offers synthetic pathways characterized by environmental compatibility and economic feasibility, while obviating the need for energy-intensive reaction conditions. Photochemistry has thus emerged as an increasingly pivotal tool for the construction of functional molecules.^16–21^ Central to this area is *E*/*Z* isomerism—a stereoisomeric process that enables innovative approaches to molecular-level structural engineering. The functional significance of *E*/*Z* isomers primarily derives from their differential sensitivity to external stimuli, a property rooted in the intrinsic molecular rearrangements accompanying conformational interchange.^22,23^ Owing to its scientific importance, photoinduced *E*/*Z* isomerization has garnered widespread research attention and exhibits versatile applications across disciplines, such as molecular photoswitching, advanced energy storage technologies, and rational drug design.^24–32^ Illustrative examples include the reversible photochromism in azobispyrazoles documented by Li *et al.*, the spatiotemporal control over pharmacological activity realized by Winssinger *et al. via* selective photoisomerization and the azobenzene-derived solar thermal fuels developed by Gupta *et al.* through efficient photon energy conversion.^33–35^ Collectively, these advances demonstrate how light-directed molecular isomerization can mediate structural reconfiguration, offering a robust methodological framework for related scientific inquiries.

In light of the considerable capability of *E*/*Z* isomerization to modulate molecular structures, its incorporation into energetic molecular scaffolds holds promise for achieving controlled structural transformations. Nevertheless, investigations into isomeric forms of energetic compounds remain relatively underdeveloped. While initial studies have verified that some energetic species, including 1,1′-azobis-1,2,3-triazole, undergo detectable *E*/*Z* photoisomerization accompanied by photochromic responses, a thorough analysis that quantifies the accompanying structural modifications in such systems is still absent.^36^ Hence, the phenomenon of isomerism in energetic materials merits deepened scientific scrutiny and methodical exploration. Research on photo-induced *E*/*Z* isomerization constitutes a viable strategy for devising new energetic compounds leveraging distinctive *E*/*Z* conformational traits, potentially creating innovative avenues for material advancement. To bridge this existing gap, we describe herein the inaugural synthesis and single-crystal structural determination of the *Z*-isomer of nitrogen-rich alkene-based energetic species 1,2-bis(tetrazol-5-yl)ethylene (H_2_bte) and present a systematic inquiry into the photo-induced *E*/*Z* isomerization mechanism operating in *E*-1,2-bis(tetrazol-5-yl)ethylene (*E*-H_2_bte). Integrated with quantum chemical computations, the structural and photophysical features of the *E*/*Z* isomers were comprehensively characterized. Furthermore, a series of metal complexes derived from the *E*/*Z* isomers were synthesized and their coordination chemistry was explored. This investigation advances the foundational knowledge of *E*/*Z* isomerization in energetic molecular systems by clarifying structural attributes of the isomeric species, thus establishing a platform for subsequent research.

## Results and discussion

**Fig. 2:**
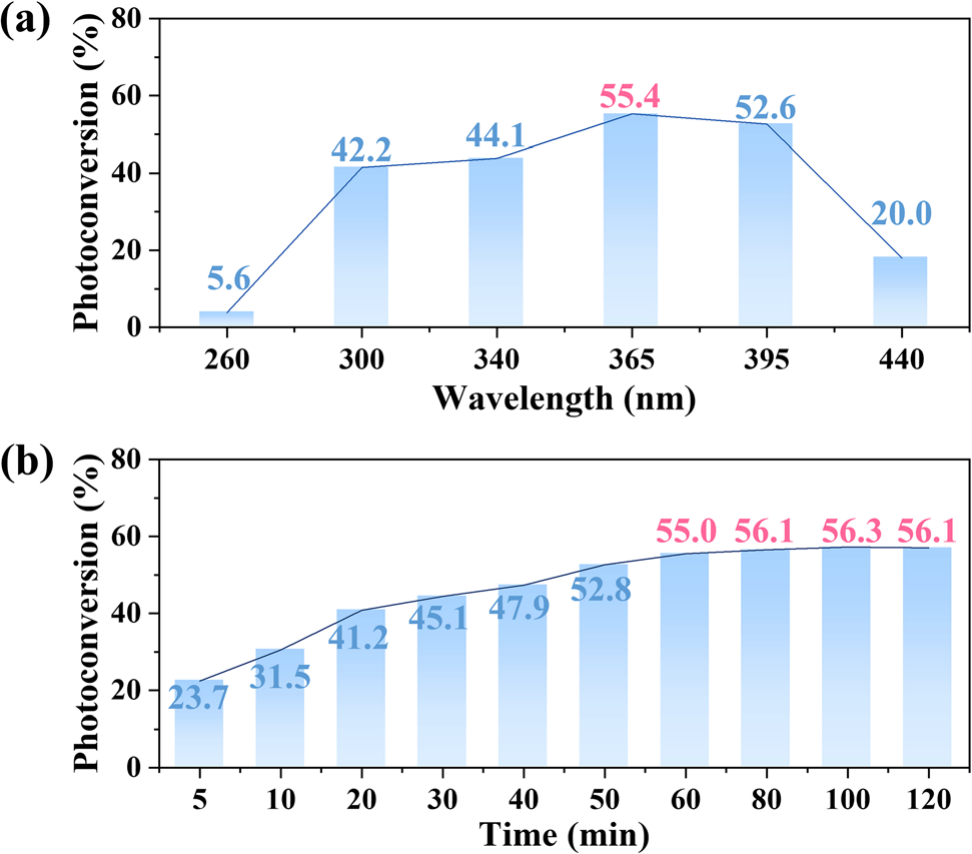
Screening conditions (10 mL, 0.05 M *E*-H_2_bte methanol solution irradiated at 25 °C). (a) Selection of wavelength. (b) Investigation of reaction time. Calculation of photoconversion: based on the ^1^H NMR results under each reaction condition, the integral of the characteristic peak of *E*-H_2_bte (7.7 ppm, CD_3_OD) was set to 1. The characteristic peak of *Z*-H_2_bte (7.2 ppm, CD_3_OD) was integrated, the integral value was denoted as *x*, and the photoconversion of *Z*-H_2_bte is *x*/(1 + *x*)%.

The initial research objective was to explore the feasibility of *E*-H_2_bte photoisomerization. Based on its solubility and considering the convenience of post-processing, we initially used methanol as the solvent for wavelength screening. To more intuitively investigate the isomerization ratio, we employed nuclear magnetic resonance (NMR) ^1^H spectra for characterization. At room temperature (25 °C), a 0.05 M solution of *E*-H_2_bte in methanol-*d*_4_ was subjected to photoisomerization under light of different wavelengths, ranging from 260 nm to 440 nm, with an irradiation power of 15 W and irradiation time of 2 h. The photoisomerization response to different wavelengths was investigated. The experimental results are shown in Fig. 2a, and indicate that *E*-H_2_bte exhibits a strong response under 365 nm and 395 nm light, with the highest isomerization ratio occurring under 365 nm light, reaching 55.4%. This demonstrates that the photoinduced *E*/*Z* isomerization of *E*-H_2_bte exhibits a pronounced wavelength dependence. Theoretical calculations reveal that *E*-H_2_bte was excited to the first excited state (S_1_), and the corresponding excitation wavelength is 273.71 nm (Table S1). This phenomenon may be influenced by the solvent.^37^ The optimal excitation wavelength is 365 nm, which can efficiently and selectively excite the molecules to the reactive S_1_ state, leading to *Z*-1,2-bis(tetrazol-5-yl)ethylene (*Z*-H_2_bte). Although the 395 nm light also selectively populates the S_1_ state, its lower molar absorption coefficient limits the overall conversion rate. In contrast, higher-energy light in the range of 260 nm to 340 nm, despite strong absorption, likely promotes excitation to higher-lying electronic states, triggering rapid internal conversion relaxation and thus suppressing efficient isomerization. After determining the irradiation wavelength of 365 nm, the other isomerization reaction conditions were screened next. The variation in the isomerization ratio with irradiation time is shown in Fig. 2b. It is evident that within one hour, the isomerization ratio exceeded 55% and reached the photo-stationary state (PSS), with the highest isomerization ratio reaching 56.3%. Subsequently, we discussed the impact of reaction temperature on the isomerization. As shown in Table 1, the photoreaction was conducted at 0 °C and −20 °C, resulting in barely any change in the isomerization ratio. Finally, *N*,*N*-dimethylformamide-*d*_7_, dimethyl sulfoxide-*d*_6_, acetone-*d*_6_ and acetonitrile-*d*_3_ were used as solvents to investigate the possibility of isomerization. The results indicate that acetone gave the highest isomerization ratio at room temperature (55.6%) among these solvents. Simultaneously, DMSO and DMF can also allow isomerization, but the conversion ratio is not satisfactory. Unfortunately, due to the poor solubility of *E*-H_2_bte in acetonitrile, significant isomerization results could not be captured. Based on the above experimental results, methanol and acetone exhibit comparable yields, while methanol is more cost-effective, which makes it the optimal solvent choice.

**Table 1:** Selection of reaction temperature and solvent^a^

Entry	Solvent	Temperature (°C)	Photoconversion^b^ (%)
1	Methanol-*d*_4_	25	55.4
2	Methanol-*d*_4_	0	55.2
3	Methanol-*d*_4_	−20	55.4
4	*N*,*N*-Dimethylformamide-*d*_7_	25	32.0
5	Dimethyl sulfoxide-*d*_6_	25	49.5
6	Acetone-*d*_6_	25	55.6
7	Acetonitrile-*d*_3_	25	Trace

a 10 mL, 0.05 M *E*-H_2_bte solution irradiated at 365 nm for 60 min.

b Calculation of photoconversion: based on the ^1^H NMR results under each reaction condition, the integral of the characteristic peak of *E*-H_2_bte (7.7 ppm, CD_3_OD) was set to 1. The characteristic peak of *Z*-H_2_bte (7.2 ppm, CD_3_OD) was integrated, the integral value was denoted as *x*, and the photoconversion of *Z*-H_2_bte is *x*/(1 + *x*)%.

**Fig. 3:**
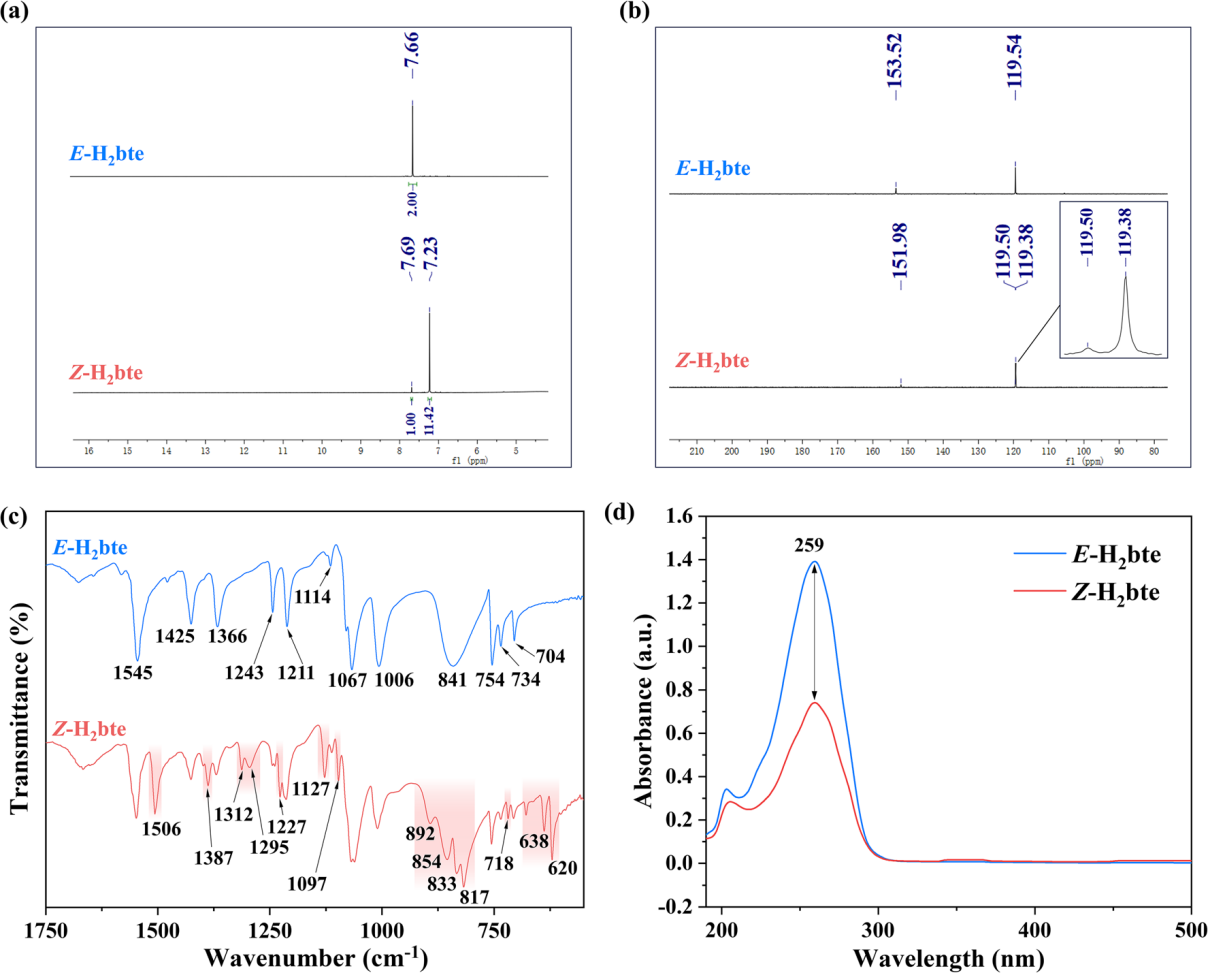
(a) ^1^H NMR in DMSO-*d*_6_ for *Z*-H_2_bte. (b) ^13^C NMR in DMSO-*d*_6_ for *Z*-H_2_bte. (c) The infrared spectra of *E*-H_2_bte and *Z*-H_2_bte. (d) The UV-vis absorption spectra of *E*-H_2_bte and *Z*-H_2_bte.

It is worth mentioning that when 365 nm irradiation was applied in dimethyl sulfoxide-*d*_6_ for one hour and the PSS was reached, we unsuccessfully attempted to realize heat-induced *Z*→*E* photoisomerization. Specifically, after reaching the PSS in dimethyl sulfoxide-*d*_6_, the system was transferred to a dark environment, then heated to 100 °C and maintained at this temperature for one hour. Verification through NMR ^1^H spectra confirmed that the ratio of *E*/*Z* isomers had barely changed and the PSS was not disrupted by increasing temperature. All the NMR spectra from the experiments are presented in the SI. This indicates that the PSS exhibits a certain degree of stability under thermal stimulation, which provides a reliable guarantee for the separation and purification processes. Therefore, it is feasible to obtain *Z*-H_2_bte *via* recrystallization. After screening several solvents, acetonitrile was chosen for recrystallization, as it effectively increases the proportion of *Z*-H_2_bte in the product. The result was confirmed by the NMR spectra (Fig. 3a and b), which showed that the *Z*-isomer constituted 91.9%. Moreover, the ^1^H and ^13^C NMR signals of the *E*→*Z* conversion shifted upfield, confirming successful promotion of the *Z*-H_2_bte isomerization. The successful isolation of the *Z*-isomer *via* recrystallization confirms that it maintains a certain degree of stability under thermal stimulation. Subsequently, an investigation was conducted into the photostability of the *Z*-isomer. Similarly, at room temperature (25 °C), a 0.05 M solution of *Z*-H_2_bte (proportion 91.9%) in methanol-*d*_4_ was subjected to photoisomerization under light of different wavelengths, ranging from 260 nm to 440 nm. The experimental results are shown in Fig. S3. Under 15 W irradiation for one hour, the proportion of *Z*-H_2_bte decreased at all wavelengths. Among these, the decrease was most significant under irradiation at 300 nm, where the proportion of *Z*-H_2_bte dropped to 61.1%, while the proportion exceeded 70% under irradiation at all remaining wavelengths. This shows that photo-induced *Z*→*E* isomerization is relatively difficult. Notably, *Z*-H_2_bte exhibits excellent stability for over three months when stored under laboratory conditions, whether as a powder sample or dissolved in deuterated reagents, with almost no change in its *E*/*Z* isomer ratio. This provides a reliable guarantee for subsequent testing and characterization.

To further validate the isomerization, multidimensional verification was conducted using infrared spectra and UV-vis spectra. The infrared spectra results are presented in Fig. 3c, where the irradiated sample displays two distinct sets of peaks. The peak located at 1545 cm^−1^ corresponds to the stretching vibrations of the N–N bond and N=N in the tetrazole ring of *E*-H_2_bte, while the corresponding peak in *Z*-H_2_bte is found at 1506 cm^−1^. The vibrational peaks at 1366, 1243, 1211 and 1067 cm^−1^ are attributed to the stretching vibrations of the C–N bond in the tetrazole ring of *E*-H_2_bte. The corresponding vibrational peaks for *Z*-H_2_bte are located at 1312, 1295, 1227, and 1127 cm^−1^. The peaks at 841 cm^−1^ and 754 cm^−1^ are associated with the in-plane and out-of-plane vibrations of the tetrazole ring in *E*-H_2_bte, while the corresponding vibrational peaks for *Z*-H_2_bte are found at 892, 854, 833, 817 and 638 cm^−1^. In addition, UV-vis spectra were used for characterization, with the results shown in Fig. 3d. There was no significant change in the maximum absorption peak before and after irradiation, and only a decrease in absorbance was observed post-irradiation. This is close to the theoretical calculated result (Tables S1 and S2).

**Fig. 4:**
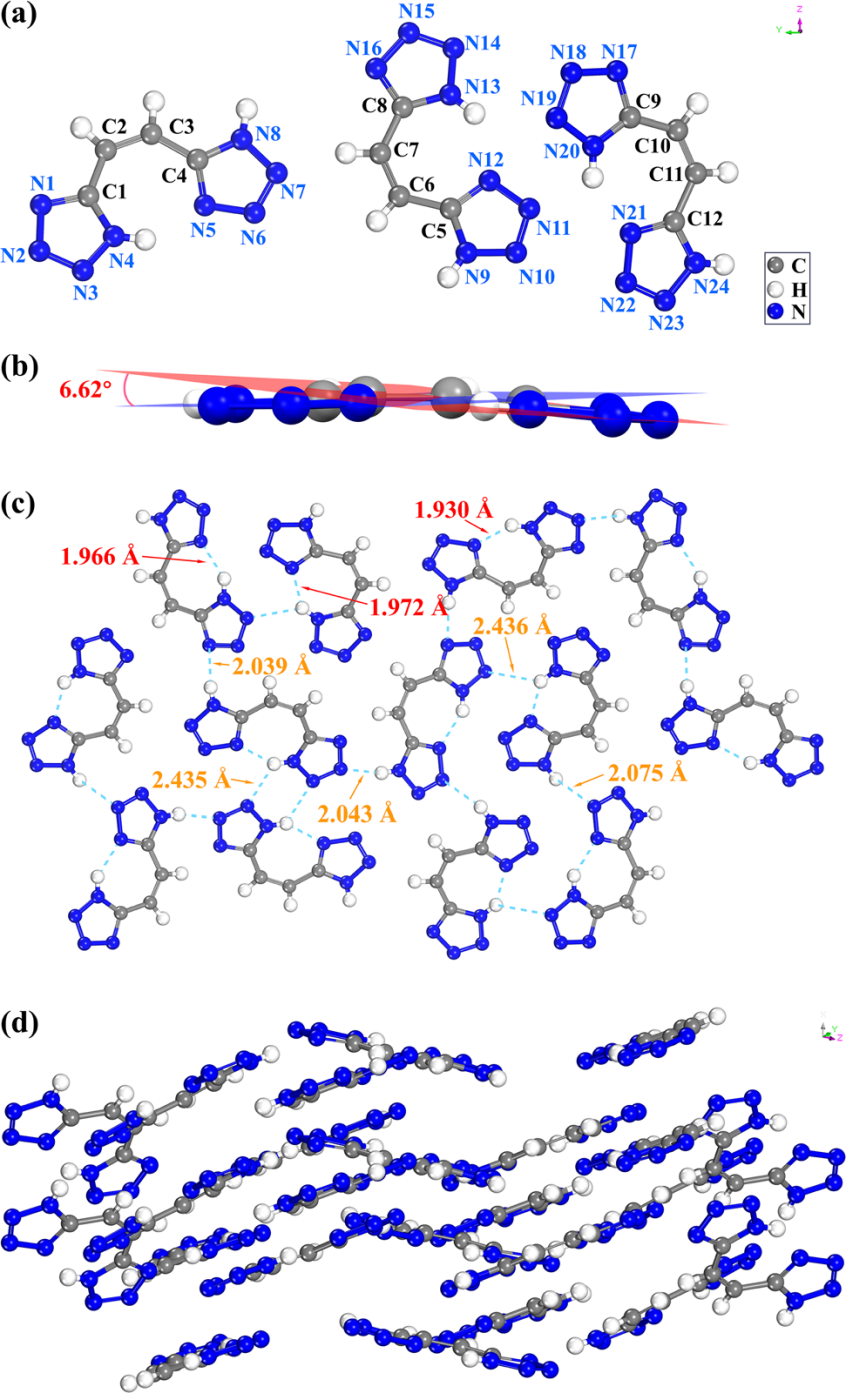
(a) Molecular structure of *Z*-H_2_bte. (b) The molecular planarity of *Z*-H_2_bte. (c) The H-bond net of *Z*-H_2_bte. (d) The crystal packing of *Z*-H_2_bte.

The crystal growth of *Z*-H_2_bte was carried out using dichloromethane. The structure of *Z*-H_2_bte was confirmed through single-crystal X-ray diffraction analysis. The molecular structure, hydrogen bonds, and crystal packing of *Z*-H_2_bte are presented in Fig. 4. *Z*-H_2_bte crystallizes in the monoclinic space group *P*2_1_/*n* with a calculated density of 1.645 g cm^−3^ at 297.6 K and contains 12 molecular moieties in the unit cell. The entire molecule is nearly planar, with torsion angles ranging from 0° to 5°. There is a 6.62° angle between the two tetrazole rings (Fig. 4b). *E*-H_2_bte crystallizes in the *P*-1 space group and triclinic crystal system with a crystal density of 1.742 g cm^−3^ at 100 K (CCDC: 1872388).^38^ Its crystal structure indicates excellent molecular planarity, with all atoms lying in the same plane, and features an intramolecular hydrogen bond of 2.137 Å. The packing mode is layered, with an interlayer distance of 2.137 Å (Fig. S4). Fig. 4c illustrates the hydrogen bond network of *Z*-H_2_bte, where it is evident that it possesses a rich array of intermolecular hydrogen bonds, ranging from 2.039 Å to 2.436 Å. Additionally, it exhibits strong intramolecular hydrogen bonds, ranging from 1.930 Å to 1.972 Å. The packing arrangement of *Z*-H_2_bte is shown in Fig. 4d, where the change in molecular planarity after isomerization leads to a modification in its packing form, resulting in the loss of the originally regular layered structure. The packing of the crystals exhibits no obvious regularity, instead adopting a relatively disordered wave-like stacking pattern. Moreover, the alteration in packing mode has a certain impact on its stability.

Thermogravimetry (TG) and differential scanning calorimetry (DSC) are key techniques for assessing thermal stability and decomposition traits, and they were employed to investigate the thermal behaviors of *E*-H_2_bte and *Z*-H_2_bte at 10 K min^−1^ under a nitrogen atmosphere. The results are shown in Fig. S45. *E*-H_2_bte has an thermal decomposition onset temperature of 265.4 °C with a peak temperature of 276.8 °C. After isomerization, *Z*-H_2_bte’s onset temperature decreases to 220.7 °C (peak: 240.8 °C). The temperature difference arises because *E*-H_2_bte maintains a highly planar conjugated structure, which enables tighter intermolecular packing and stronger π–π stacking as well as van der Waals forces. *Z*-H_2_bte has a sterically distorted structure that disrupts dense intermolecular packing and weakens intermolecular interactions, which in turn lowers its decomposition temperature.

The physicochemical and energetic properties of *E*-H_2_bte and *Z*-H_2_bte were measured and calculated. The solid-phase heat of formation of *E*-H_2_bte and *Z*-H_2_bte was calculated by using the Gaussian 09 (Revision D.01) program. Owing to the tetrazole present in their molecular structures, both *E*-H_2_bte and *Z*-H_2_bte possess very positive heats of formation; the outcome values are 612.57 kJ mol^−1^ and 620.09 kJ mol^−1^, respectively. The solid-phase heat of formation of *Z*-H_2_bte is slightly higher than that of *E*-H_2_bte. On this basis, the detonation performances were calculated using EXPLO5 (version 6.02) software. The results indicate that the detonation performance of *Z*-H_2_bte (*D* = 8313 m s^−1^, *P* = 23 GPa) was lower than that of *E*-H_2_bte (*D* = 8725 m s^−1^, *P* = 26 GPa) after isomerization. Meanwhile, a standard BAM fall hammer and BAM friction tester were employed to measure the mechanical sensitivities. As expected, the sensitivity of *Z*-H_2_bte (IS = 17.5 J, FS = 144 N) was higher than that of *E*-H_2_bte (IS = 22.5 J, FS = 252 N). All the physicochemical and energetic properties are listed in Table 2.

**Table 2:** Physicochemical properties of *E*-H_2_bte and *Z*-H_2_bte

	*T*_d_ a (°C)	*ρ* (g cm^−3^)	Δ*H*_f_^d^ (kJ mol^−1^)	*D* e (m s^−1^)	*P* f (GPa)	IS^g^ (J)	FS^h^ (N)
*E*-H_2_bte	265.4	1.720^b^	612.57	8725	26	22.5	252
*Z*-H_2_bte	220.7	1.645^c^	620.09	8313	23	17.5	144

a Thermal decomposition temperature (onset) under nitrogen gas (DSC, 10 °C min^−1^).

b Density – gas pycnometer at 298 K (ref. 38).

c Density measured *via* single-crystal X-ray diffraction at 298 K.

d Calculated heat of formation.

e Calculated detonation velocity.

f Calculated detonation pressure.

g Impact sensitivity.

h Friction sensitivity.

**Fig. 5:**
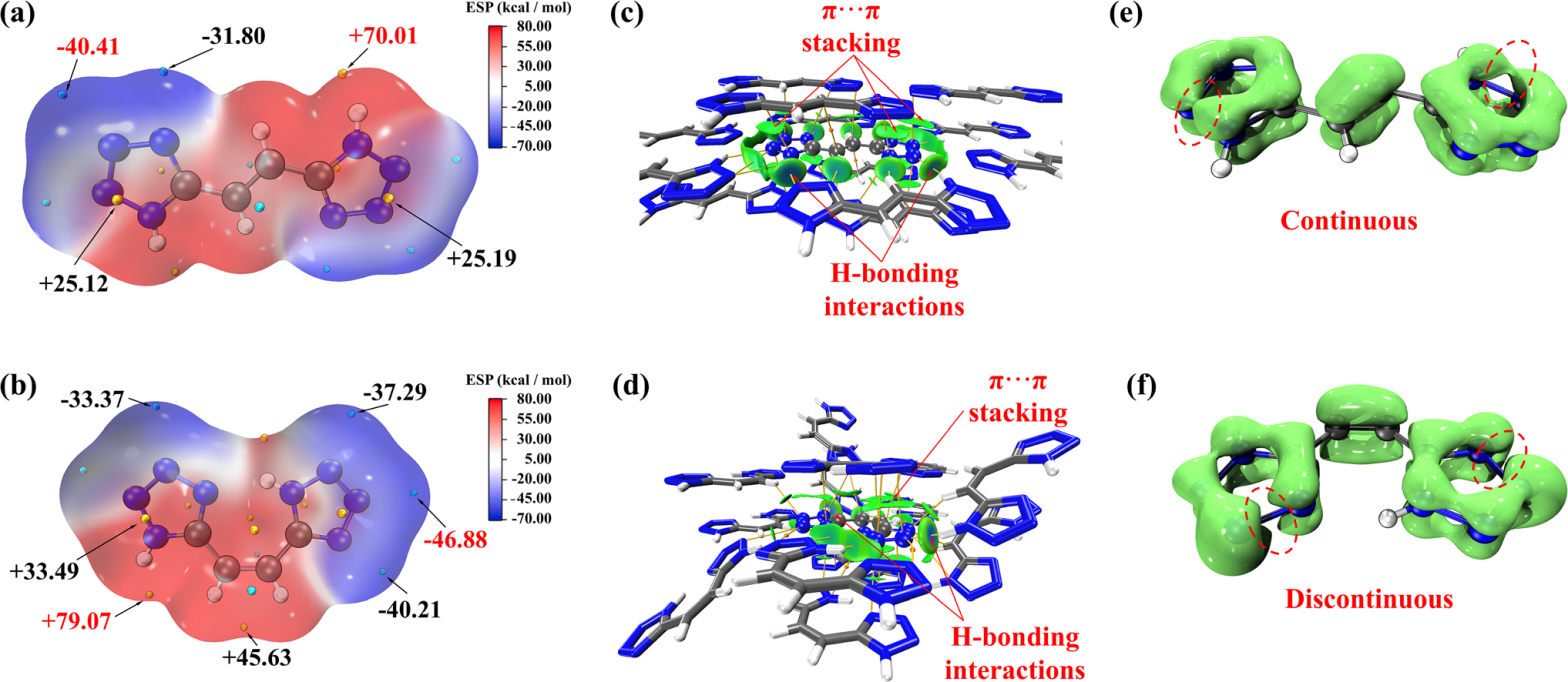
(a and b) Surface electrostatic potentials of *E*-H_2_bte and *Z*-H_2_bte. (c and d) Noncovalent-interaction analysis of *E*-H_2_bte and *Z*-H_2_bte. (e and f) LOL-π electron delocalization isosurface maps of *E*-H_2_bte and *Z*-H_2_bte.

In order to better understand the changes in properties brought about by isomerization, the electrostatic potentials (ESP) of *E*-H_2_bte and *Z*-H_2_bte were calculated.^39^ As depicted in Fig. 5a and b, the blue regions represent the electronegative areas, while the red regions are regarded as electropositive areas. Generally, the stronger ESP surfaces often result in higher sensitivities. Compared to *Z*-H_2_bte, *E*-H_2_bte exhibits red and blue alternating ESP mapped surfaces. Moreover, range between the minima and maxima of the ESP for molecule *E*-H_2_bte (−40.41–70.01 kcal mol^−1^) is narrower than that of *Z*-H_2_bte (−46.88 kcal mol^−1^–79.07 kcal mol^−1^).^40^ All the results indicate that *E*-H_2_bte is more stable. In the meantime, the noncovalent interaction (NCI) analysis of both compounds is calculated and discussed.^41^ As shown in Fig. 5c and d, *E*-H_2_bte exhibits extensive π⋯π stacking in the region between two layers of the molecules, as indicated by the green areas in the figure. Concurrently, hydrogen bonding interactions are present between the molecules, as shown in the blue areas of the figure. In the case of *Z*-H_2_bte, the complex stacking arrangement following isomerization results in reduced π⋯π stacking between the molecules. At the same time, the hydrogen bonding interactions between the molecules are also diminished compared to those in *E*-H_2_bte. This confirms the observed increase in sensitivity of *Z*-H_2_bte after isomerization. LOL-π (π-electron localized orbital locator) serves as a crucial criterion for evaluating the stability of compounds in terms of π-electron distribution.^42^ To further evaluate the thermal stability of *E*-H_2_bte and *Z*-H_2_bte, the π-electron isosurfaces of *E*-H_2_bte and *Z*-H_2_bte were calculated and visualized (Fig. 5e and f). The results indicate that the LOL-π isosurfaces of the tetrazole rings in *E*-H_2_bte are continuous. In contrast, under the same isosurface conditions, the LOL-π isosurfaces of the tetrazole rings in *Z*-H_2_bte are discontinuous. This suggests that the greater areas of π-electron distributions contribute to the superior thermostability and aromaticity of *E*-H_2_bte.

**Fig. 6:**
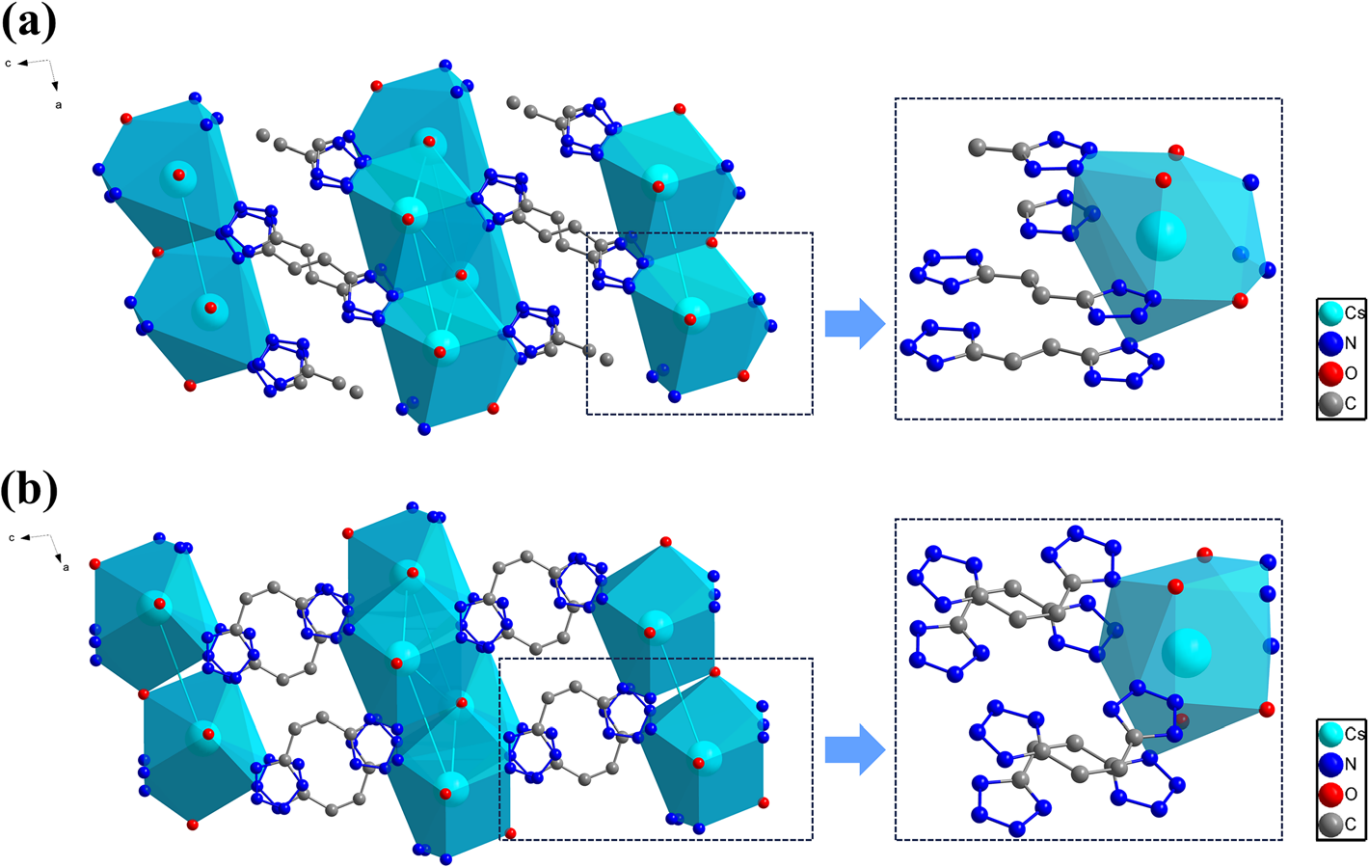
(a) 2-D packing diagram of 3 representing the connectivity of the *E*-Hbte linker and Cs(i) coordination sphere. (b) 2-D packing diagram of 4 representing the connectivity of the *Z*-Hbte linker and Cs(i) coordination sphere.

Four metal complexes (1: *E*-NaHbte, 2: *Z*-NaHbte, 3: *E*-CsHbte and 4: *Z*-CsHbte) based on both isomeric ligands were synthesized, and single-crystal structures were obtained of them. Notably, coordination improved the molecular planarity of the *Z*-ligand. Compared with *Z*-H_2_bte, the dihedral angles between the two tetrazole rings were reduced, with the smallest angle being only 2.03° (Fig. S5) and that of 4 being reduced to 5.63° (Fig. S6). Fig. 6 presents the 2-D packing diagrams of 3 and 4. Both complexes crystallize in the triclinic crystal system, belonging to the *P*-1 space group. In the structures of 3 and 4, the cesium atoms form coordination bonds with nitrogen atoms of the ligands and oxygen atoms of water molecules, thereby constructing two distinct coordination polyhedra. Structural analyses confirm that 3 features a CsN_10_O_3_ coordination environment, with coordination bond lengths ranging from 3.232 Å to 3.530 Å, while 4 exhibits a CsN_9_O_4_ coordination environment, with bond lengths spanning 3.294 Å to 3.529 Å. For 3, both tetrazole rings of the Hbte ligand coordinate to cesium ions *via* nitrogen atoms, exhibiting dipodal coordination connectivity. Hbte linkers coordinated to terminal cesium ions connect along the crystallographic *c*-axis, forming a 1-D chain structure. These 1-D chains are interconnected along the *b*-axis by another set of Hbte linkers coordinated to bridging cesium ions, constructing a 2-D sheet structure extending along the bc plane (Fig. 6a). 4 also adopts the same connection and packing modes (Fig. 6b). In addition, 1 and 2 share the same coordination mode (Fig. S7). Both complexes feature a monodentate coordination mode and an octahedral NaNO_5_ coordination environment around the terminal sodium ions.^43^ All of the units are linked to one another in a dipodal planar manner. Therefore, among these four complexes, upon coordination with the same metal ion, the packing and connection modes are found to be analogous and their coordination modes are likely to be identical (1 and 2). The only difference in their crystal packing modes between each pair resides in the orientation of the tetrazole rings within the Hbte ligands, which is dependent on the *E*/*Z* configuration of the parent isomers. This will be critical for photoresponsive coordination polymer design in future work.

## Conclusions

In summary, this work systematically explores the photoinduced *E*/*Z* isomerization of the nitrogen-rich alkene energetic molecule 1,2-bis(tetrazol-5-yl)ethylene (H_2_bte) and its coordination chemistry, delivering key findings. Optimized 365 nm UV irradiation followed by recrystallization enabled efficient *E*→*Z* isomerization, yielding phase-pure *Z*-1,2-bis(tetrazol-5-yl)ethylene (*Z*-H_2_bte) (91.9%). This is the first reported single-crystal structure of *Z*-H_2_bte, revealing a non-planar configuration *versus* the planar *E*-isomer. The structural disparity induces distinct hydrogen-bonding networks and crystal packing. The experimental results also indicate that a set of complexes formed by coordination of *E*/*Z* isomeric ligands with the same metal ion exhibits analogous packing and connection modes. This research fills the structural characterization gap of *Z*-isomers in nitrogen-rich alkene energetic compounds, advancing insights into *E*/*Z* isomerism.

## Experiment section

The products under consideration exhibit significant hazards, particularly due to the potential for explosions under specific conditions. Despite the absence of any difficulties or accidents in our current handling procedures, these experiences were limited to small-scale operations. As such, it is essential to establish stringent safety protocols in anticipation of larger-scale handling. Comprehensive safety training must be implemented, with mandatory use of personal protective equipment, including protective coats, leather gloves, and face shields. Furthermore, it is crucial to avoid mechanical actions that could compromise the integrity of the products, such as scratching or scraping.

All material characterization methods and experimental details are provided in the SI. The *E*-1,2-bis(tetrazol-5-yl)ethylene (*E*-H_2_bte) was synthesized according to the literature.^38^ The synthesis of *Z*-1,2-bis(tetrazol-5-yl)ethylene (*Z*-H_2_bte) was performed in photochemical reaction equipment, with additional details provided in the SI. At room temperature, a 0.05 M solution of *E*-H_2_bte in a quartz glass reaction tube was irradiated at a specific wavelength. After the reaction was completed, the solution was concentrated under vacuum to yield a pale yellow powder. This powder was recrystallized from acetonitrile; after cooling and filtration, the filtrate was concentrated under vacuum to remove the solvent, leaving a white powder of high-purity *Z*-H_2_bte, yield: 51.46%. In addition, the sodium (1 and 2), caesium (3 and 4) complexes of both *E*-H_2_bte and *E*-H_2_bte were prepared in high yields (yield for 1: 88.12%; 2: 85.42%; 3: 68.22%; 4: 61.71%). In a typical experiment, an aqueous solution of *E*-H_2_bte or *Z*-H_2_bte was heated to 90 °C. The corresponding metal-salt solution was added dropwise, and the mixture was maintained at 90 °C for 20 min. After the reaction was completed, the mixture was filtered while hot. The filtrate was allowed to evaporate to yield the product. Detailed synthetic procedures and characterization data are provided in the experimental section of the SI.

## Author contributions

Junhao Shi: data curation, investigation, methodology and manuscript writing. Sicheng Liao: conceptualization, investigation, funding acquisition. Tianyu Jiang: implementation of the computer code and supporting algorithms. Shiliang Huang and Kangcai Wang: X-ray data collection and methodology. Wenquan Zhang: conceptualization, investigation, supervision, funding acquisition, manuscript writing and editing.

## Conflicts of interest

The authors declare there are no competing financial interests.

## Supplementary Material

RA-016-D5RA09255H-s001

RA-016-D5RA09255H-s002

## Data Availability

CCDC 2474044, 2474046, 2474047 and 2474069 contain the supplementary crystallographic data for this paper.^44*a*–*d*^ The data supporting this article have been included as part of the supplementary information (SI). Supplementary information: partial experimental and computational results, crystallographic data, NMR spectra, powder X-ray diffraction patterns, infrared spectrum, and TG/DSC results. See DOI: https://doi.org/10.1039/d5ra09255h.
