# Supplementary material for: Synthesis and structural characterization of the Z-isomer of 1,2-bis(tetrazol-5-yl)ethylene (H2bte) via photoinduced isomerization
Source: RSC Adv. 2026 Jan 13;16(3):2724–31. doi: 10.1039/d5ra09255h (PMC12797301; doi:10.1039/d5ra09255h)
Supplement: RA-016-D5RA09255H-s001 [file RA-016-D5RA09255H-s001.pdf]

Supplementary Information (SI) for RSC Advances.
This journal is © The Royal Society of Chemistry 2025

Supplementary Information

Synthesis and Structural Characterization of the *Z*-Isomer of 1,2-Bis(tetrazol-5-yl)ethylene (H_2bte) via Photoinduced Isomerization

Junhao Shi[1,†], Sicheng Liao[1,†], Shiliang Huang[1], Tianyu Jiang[1], Kangcai Wang[1], Wenquan Zhang[1,]*

[1] Institute of Chemical Materials, China Academy of Engineering Physics, Mianyang 621999, China.

† These authors contributed equally.

Corresponding Author

*Email: zhangwq-cn@caep.cn (Wenquan Zhang).

Contents

1. Experimental section.......... S3

1.1 Methods and materials S3

1.2 Experimental phenomenon.......... S5

1.3 Experimental results supplement.......... S5

2 Theoretical calculations S6

3. Crystallographic data.......... S7

4. NMR Spectra S11

5. Powder X-ray diffraction patterns S28

6. Infrared spectrum.......... S29

7. TG/DSC curve.......... S29

7. References.......... S29

1. Experimental section

1.1 Methods and materials

All used chemical reagents and solvents were of analytical grade and were used as supplied, without purification. The light reaction equipment (RLH-18CU) was acquired from Company Beijing Roger Technology Co., LTD. The experimental equipment is shown in Figure S1. All reactions were carried out in quartz reaction tubes. Lamp sheets of different wavelengths were used to irradiate from the bottom of the reaction tube. The reaction equipment also included temperature control, cooling circulation, power adjustment, and magnetic stirring control.

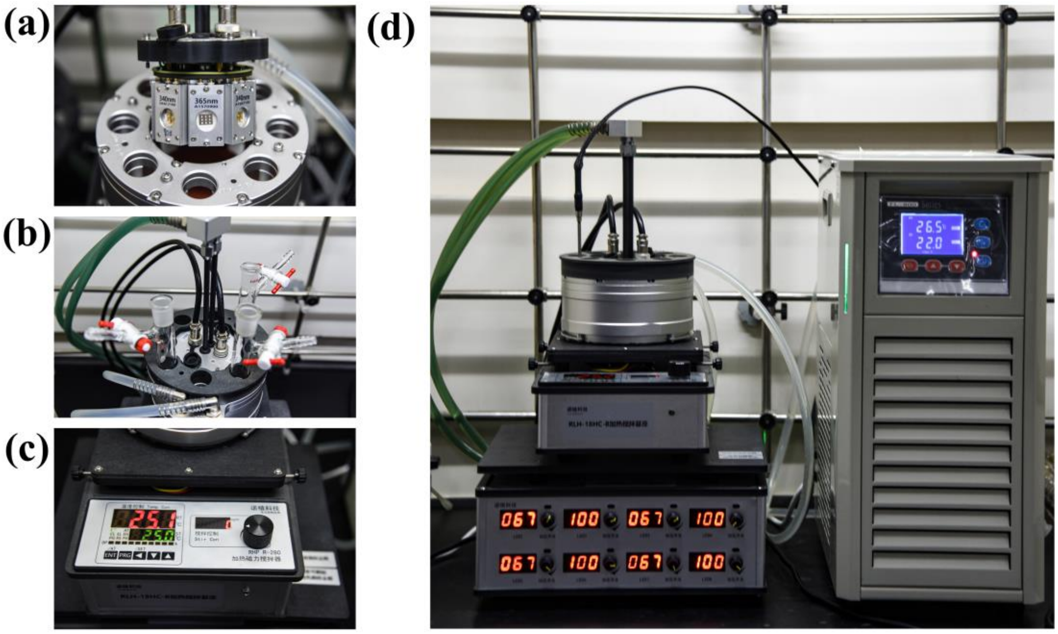

Figure S1. (a) Schematic diagram of a lamp slide. (b)Schematic of the reaction in a quartz tube. (c) Temperature control and magnetic stirring control. (d) Equipment diagram.

The Bruker Advance 400 NMR spectrometer was used at 400 MHz and 100 MHz, respectively, with deuterated dimethyl sulfoxide-d_6 and methanol-d_4 et al. as the solvent, the ^{1}H NMR and ^{13}C NMR spectra of the samples were tested. X-ray single-crystal diffraction data were collected on an Oxford Xcalibur 3 diffractometer with Cu K_α or Mo K_α radiation (λ = 1.54178 Å). The direct method and full-matrix least-squares method on F2 contained in the Olex2 program package were used to resolve and refine the structures. The powder X-ray diffraction patterns were collected on a Bruker D8 Advance, and the 2θ range measured was 5°–50° with steps of 0.02°/0.1 s. Thermal property measurements were conducted using a TGA/DSC Mettler Toledo calorimeter equipped with an auto-cooling accessory. The samples were heated at a rate of 10 K min^{-1} under a nitrogen atmosphere. PerkinElmer Spectrum two Fourier transform infrared spectrometer was used, andpotassium bromide was used to grind and press tablets at 4000 cm^{-1}–400 cm^{-1} range test. Impact and friction sensitivities were measured at ambient temperature by a standard BAM falling hammer and a BAM friction tester.

Localized orbital locator-π (LOL-π) maps, electrostatic potential (ESP) surfaces and non-covalent interaction (NCI) analyses were calculated and analyzed by using Gaussian 09 (Revision D.01), Multiwfn (version 3.8) and Visual Molecular Dynamics (VMD, version 1.9.2) program suites [S1-S4].

Preparation of *Z*-H_2bte. 0.164 g *E*-H_2bte (1 mmol) was dissolved in 20 mL methanol. The solution was irradiated at 365 nm for 1 hour. Vacuum concentration yielded a pale yellow powder. Recrystallization from acetonitrile gave a white powder of high-purity *Z*-H_2bte. Yield: 51.46%. IR (KBr, cm^{-1}): 3043, 1666, 1548, 1506, 1425, 1387, 1369, 1312, 1295, 1239, 1227, 1213, 1127, 1061, 1097, 1010, 892, 854, 833, 817, 754, 718, 638, 620, cm^{-1}. ^{1}H NMR (400 MHz, DMSO-d_6) δ: 7.23, ^{13}C NMR (100 MHz, DMSO-d_6) δ: 151.98, 119.38. Elemental analysis for $C_4H_4N_8$ (164.13): C, 29.27; H, 2.46; N, 68.27; Found: C 29.11, H 2.61, N 67.13.

General process preparation of Comp. 1-4. 1 mmol *E*-H_2bte or *Z*-H_2bte was suspended in water, the suspension was heated to 90 °C, the 1 mmol appropriate inorganic metal salt (sodium hydroxide, cesium carbonate, copper (II) acetate, lead (II) acetate)was added, and the mixture was maintained at this temperature for 20 min. After the reaction, the mixture was filtered while hot; the filtrate was collected and left to evaporate at ambient temperature, yielding the product.

Comp. 1: IR (KBr, cm^{-1}): 3467, 2232, 1643, 1566, 1498, 1407, 1217, 1201, 1150, 1134, 1071, 1035, 1020, 964, 775, 753, 643, 531, cm^{-1}. ^{1}H NMR (400 MHz, DMSO-d_6) δ: 7.47, ^{13}C NMR (100 MHz, DMSO-d_6) δ: 156.92, 118.95. Elemental analysis for Comp. 1: C, 18.49; H, 3.89; N, 43.12. Yield: 88.12%.

Comp. 2: IR (KBr, cm^{-1}): 3434, 2231, 1643, 1568, 1505, 1409, 1340, 1324, 1228, 1211, 1195, 1143, 1103, 1041, 1018, 966, 935, 807, 775, 756, 638, 622, 538, cm^{-1}. ^{1}H NMR (400 MHz, DMSO-d_6) δ: 7.12, ^{13}C NMR (100 MHz, DMSO-d_6) δ: 154.81, 116.58. Elemental analysis for Comp. 2: C, 17.71; H, 3.78; N, 42.73. Yield: 85.42%.

Comp. 3: IR (KBr, cm^{-1}): 3353, 2350, 1886, 1641, 1576, 1482, 1466, 1412, 1241, 1217, 1191, 1147, 1117, 1107, 1084, 1042, 1026, 966, 842, 764, 734, 486, cm^{-1}. ^{1}H NMR (400 MHz, DMSO-d_6) δ: 7.35, ^{13}C NMR (100 MHz, DMSO-d_6) δ: 159.81, 119.12. Elemental analysis for Comp. 3: C, 11.15; H, 0.92; N, 25.70. Yield: 68.22%.

Comp. 4: IR (KBr, cm^{-1}): 3400, 3049, 1970, 1881, 1658, 1530, 1458, 1415, 1337, 1211, 1125, 1094, 1027, 967, 819, 764, 646, 520, cm^{-1}. ^{1}H NMR (400 MHz, DMSO-d_6) δ: 6.62, ^{13}C NMR (100 MHz, DMSO-d_6) δ: 158.76, 119.08. Elemental analysis for Comp. 4: C, 10.28; H, 1.19; N, 24.11. Yield: 61.71%.

1.2 Experimental phenomenon

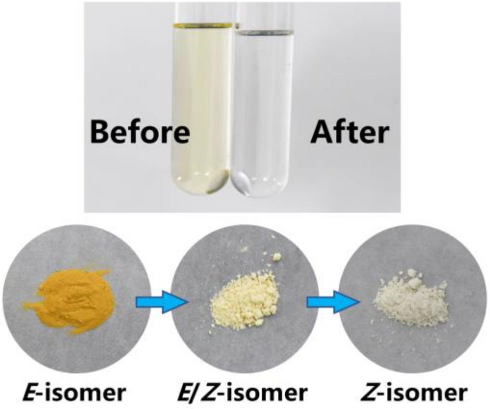

Figure S2. The state of the reaction mixture before and after the reaction and the photographs of the relevant reactants.

1.3 Experimental results supplement

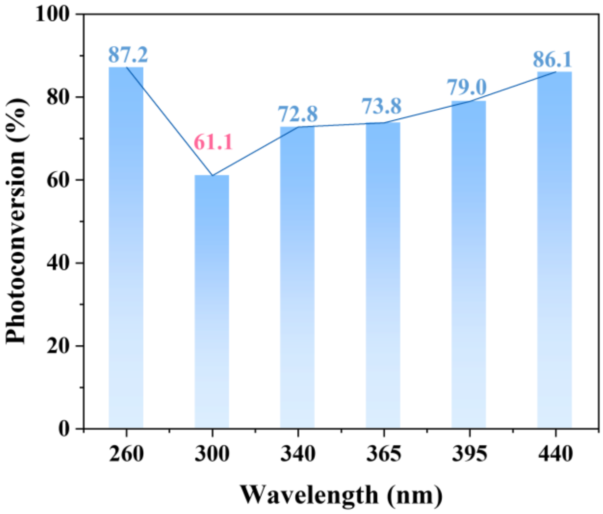

Figure S3. Photoconversion results of *Z*-H_2bte under irradiation at different wavelengths.

2 Theoretical calculations

a. Heats of formation

The gas-phase enthalpies of the *E*-H_2bte or *Z*-H_2bte are obtained by using the atomization method with the G4(MP2)_6x method (J. Chem. Theory Comput., 2011,7, 112-120). The solid-state enthalpy of formation was estimated by subtracting the heat of sublimation from gas-phase heat of formation. The heat of sublimation can be estimated with Trouton's rule according to equation 1[S5]:

$$\Delta H_{sub} = 188/J{\cdot}mol^{-1}K^{-1} \times T \qquad \text{(equation 1)}$$

where T represents either the melting point or the decomposition temperature when no melting occurs prior to decomposition.

b. Excitation energy, wavelengths and oscillator strengths

The molecular geometries were optimized at B3LYP-D3BJ/6-311+G** level of theory using PCM solvation model in methanol. The vertical electronic excitation energies for *E*-H_2bte or *Z*-H_2bte were calculated by timedependent density functional theory (TDDFT). The lowest-lying singlet excited states were considered at pbe1pbe/def2TZVP level of theory with SMD solvation model in methanol (**Table S1** and **Table S2**).

Table S1 Calculated excitation energy, excitation wavelengths (λ) and oscillator strengths (*f*) of *E*-H_2bte in MeOH.

	Energy (eV)	λ (nm)	*f*
HOMO→LUMO	4.5298	273.71	0.90620
HOMO-1→LUMO	5.1246	241.94	0.00000
HOMO-2→LUMO	5.2532	236.02	0.00410

Table S2 Calculated excitation energy, excitation wavelengths (λ) and oscillator strengths (*f*) of *Z*-H_2bte in MeOH.

	Energy (eV)	λ (nm)	*f*
HOMO→LUMO	4.5907	270.08	0.60420
HOMO-1→LUMO	5.1328	241.55	0.00150
HOMO-2→LUMO	5.3490	231.79	0.00000

3. Crystallographic data

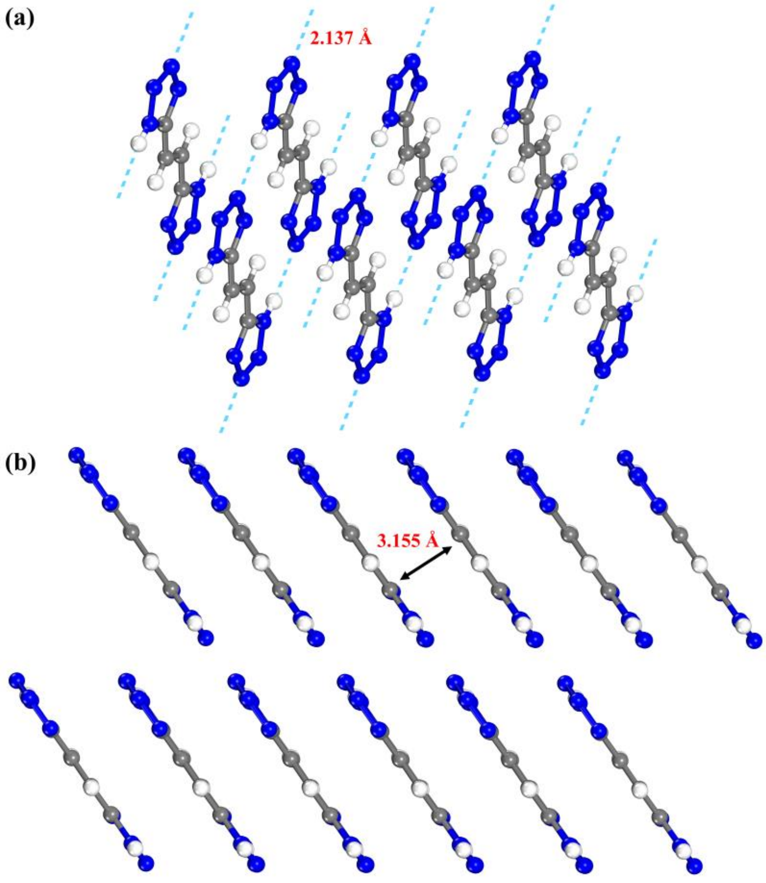

Figure S4. (a) The molecular planarity of *E*-H_2bte. (b) The crystal packing of *E*-H_2bte.

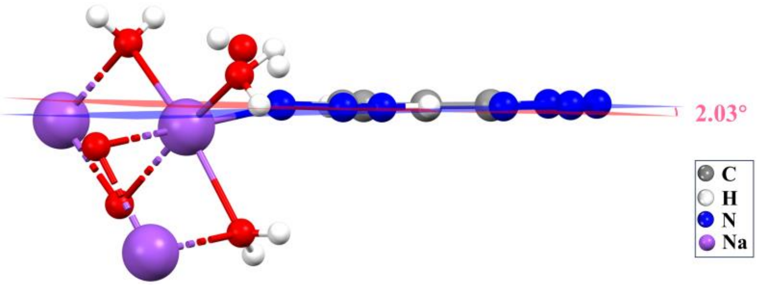

Figure S5. The molecular planarity of Comp. 2.

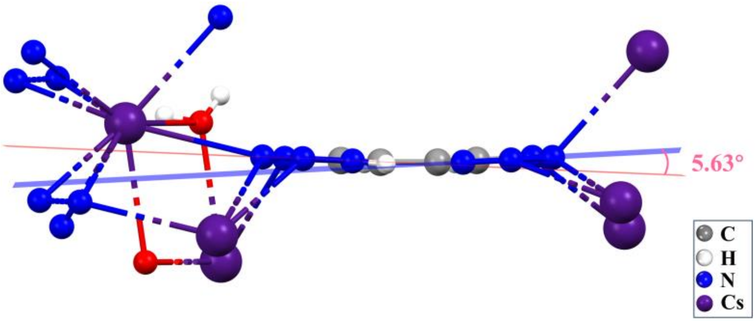

Figure S6. The molecular planarity of Comp.4.

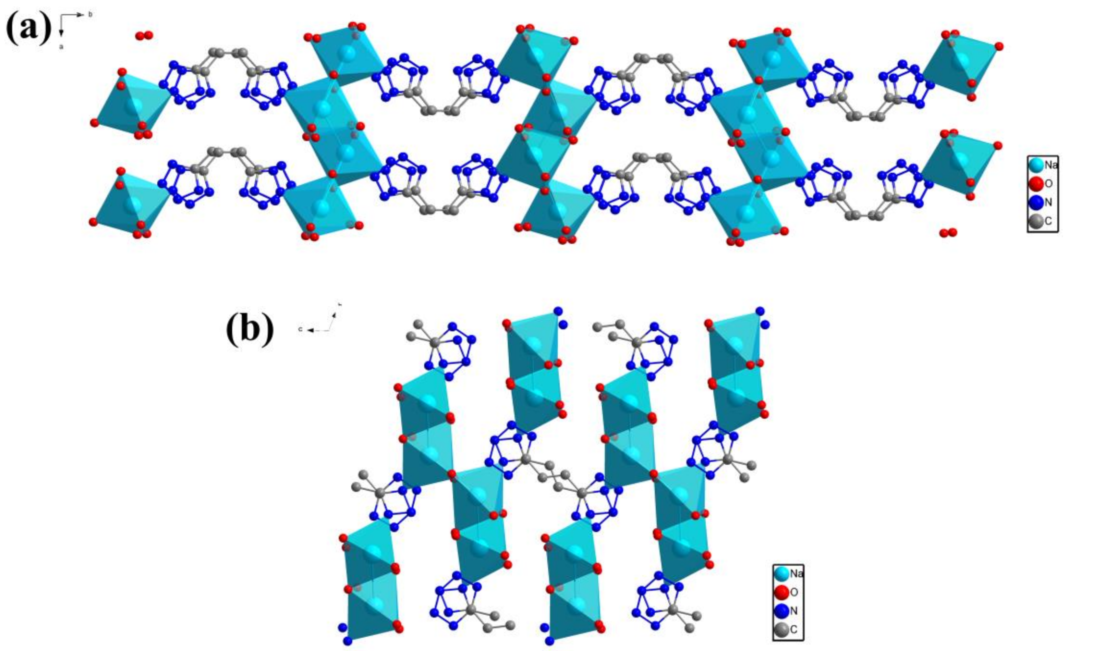

Figure S7. (a) 2-D packing diagram of Comp. 2 representing the connectivity of *Z*-Hbte linker and Na(I) coordination sphere. (b) 2-D packing diagram of Comp. 1 representing the connectivity of *E*-bte linker and Na(I) coordination sphere.

Table S3. Crystal data and structure refinement for *Z*-H_2bte and Comp. 2.

	Z-H_2bte	Comp. 2
Empirical formula	$C_4H_4N_8$	$C_4H_9N_8NaO_3$
Formula weight	164.15	240.18
Temperature/K	297.6(3)	292.78(10)
Crystal system	*monoclinic*	*monoclinic*
Space group	$P2_1/n$	$P2_1/c$
a/Å	3.79830(10)	5.7806(2)
b/Å	20.4558(2)	24.5527(5)
c/Å	25.5993(4)	7.2544(2)
α/°	90	90
β/°	90.1640(10)	97.077(2)
γ/°	90	90
V/Å^3	1988.99(6)	1021.77(5)
Z	12	4
ρ_{calc}/g·cm^{-3}	1.645	1.561
μ/mm^{-1}	1.054	1.488
F(000)	1008.0	496.0
Crystal size/mm^3	0.4 × 0.2 × 0.1	0.19 × 0.12 × 0.06
Radiation	Cu K_α (λ = 1.54178)	Mo K_α (λ = 1.54184)
2θ range for data collection/°	6.906 to 148.958	7.2 to 154.832
Index ranges	$-4 \leq h \leq 4$, $-25 \leq k \leq 16$, $-31 \leq l \leq 31$	$-7 \leq h \leq 7$, $-30 \leq k \leq 24$, $-30 \leq k \leq 24$
Reflections collected	14575	7537
Independent reflections	4001[R_{int} = 0.0617, R_{sigma} = 0.0528]	2133[R_{int} = 0.0563, R_{sigma} = 0.0487]
Data/restraints/parameters	4001/0/325	2133/1/177
Goodness-of-fit on F^2	1.030	1.086
Final R indexes [I>=2σ (I)]	R_1 = 0.0538, wR_2 = 0.1513	R_1 = 0.0426, wR_2 = 0.1077
Final R indexes [all data]	R_1 = 0.0754, wR_2 = 0.2073	R_1 = 0.0625, wR_2 = 0.1277
Largest diff. peak/hole/eÅ^{-3}	0.31/-0.45	0.26/-0.32
CCDC	2474069	2474047

Table S4. Crystal data and structure refinement for Comp. 3 and Comp.4.

	Comp. 3	Comp. 4
Empirical formula	$C_4H_5CsN_8O$	$C_4H_5CsN_8O$
Formula weight	314.07	314.07
Temperature/K	293.15	293(2)
Crystal system	*triclinic*	*triclinic*
Space group	*P*-1	*P*-1
a/Å	5.7931(2)	6.1358(2)
b/Å	7.3909(2)	6.9761(2)
c/Å	10.8114(3)	11.3456(2)
α/°	98.620(2)	99.452(2)
β/°	95.588(2)	102.405(2)
γ/°	92.568(2)	95.643(2)
V/Å^3	454.67(2)	463.34(2)
Z	2	2
ρ_{calc}/g·cm^{-3}	2.294	2.251
μ/mm^{-1}	31.694	31.101
F(000)	296.0	296.0
Crystal size/mm^3	0.26 × 0.13 × 0.11	0.18 × 0.11 × 0.06
Radiation	Mo K_α (λ = 1.54184)	Mo K_α (λ = 1.54184)
2θ range for data collection/°	8.318 to 155.35	8.128 to 155.362
Index ranges	$-6 \le h \le 7$, $-9 \le k \le 8$, $-12 \le l \le 13$	$-7 \le h \le 7$, $-8 \le k \le 8$, $-14 \le l \le 7$
Reflections collected	5144	4492
Independent reflections	1814[R_{int} = 0.0591, R_{sigma} = 0.0568]	1810[R_{int} = 0.0539, R_{sigma} = 0.0519]
Data/restraints/parameters	1814/2/135	1810/1/140
Goodness-of-fit on F^2	1.064	1.114
Final R indexes [I>=2σ (I)]	R_1 = 0.0538, wR_2 = 0.1430	R_1 = 0.0472, wR_2 = 0.1238
Final R indexes [all data]	R_1 = 0.0572, wR_2 = 0.1507	R_1 = 0.0521, wR_2 = 0.1406
Largest diff. peak/hole/eÅ^{-3}	1.43/-1.98	1.21/-1.57
CCDC	2474044	2474046

4. NMR Spectra

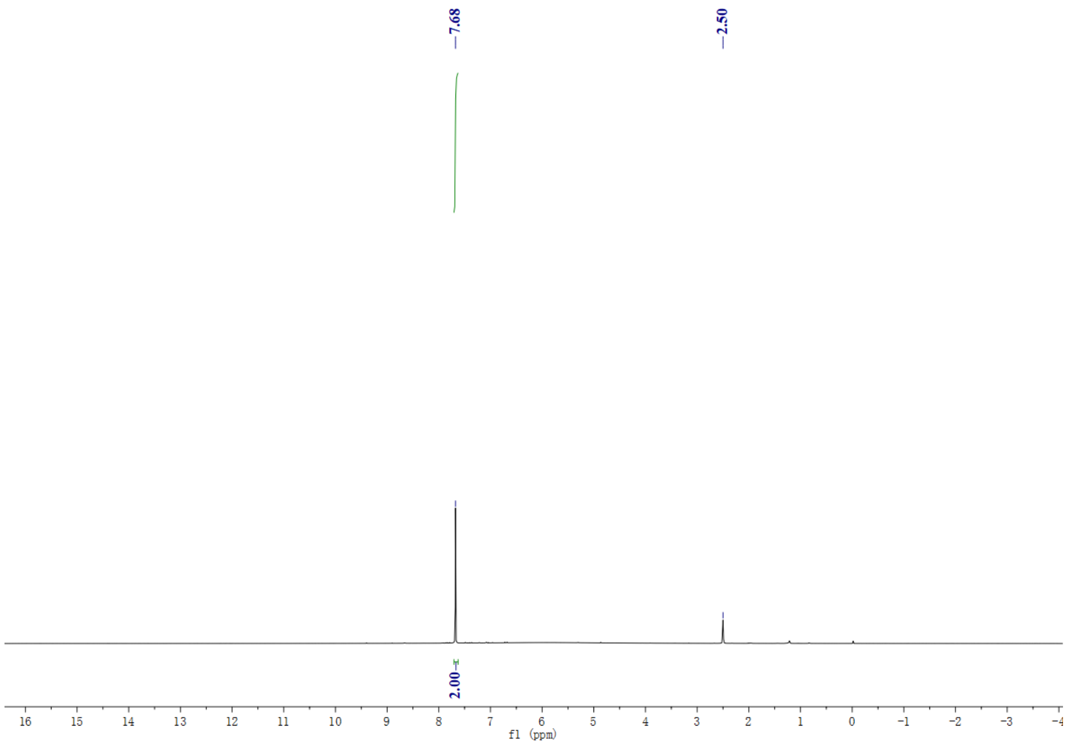

Figure S8. ^{1}H NMR in DMSO-d_6 for *E*-H_2bte.

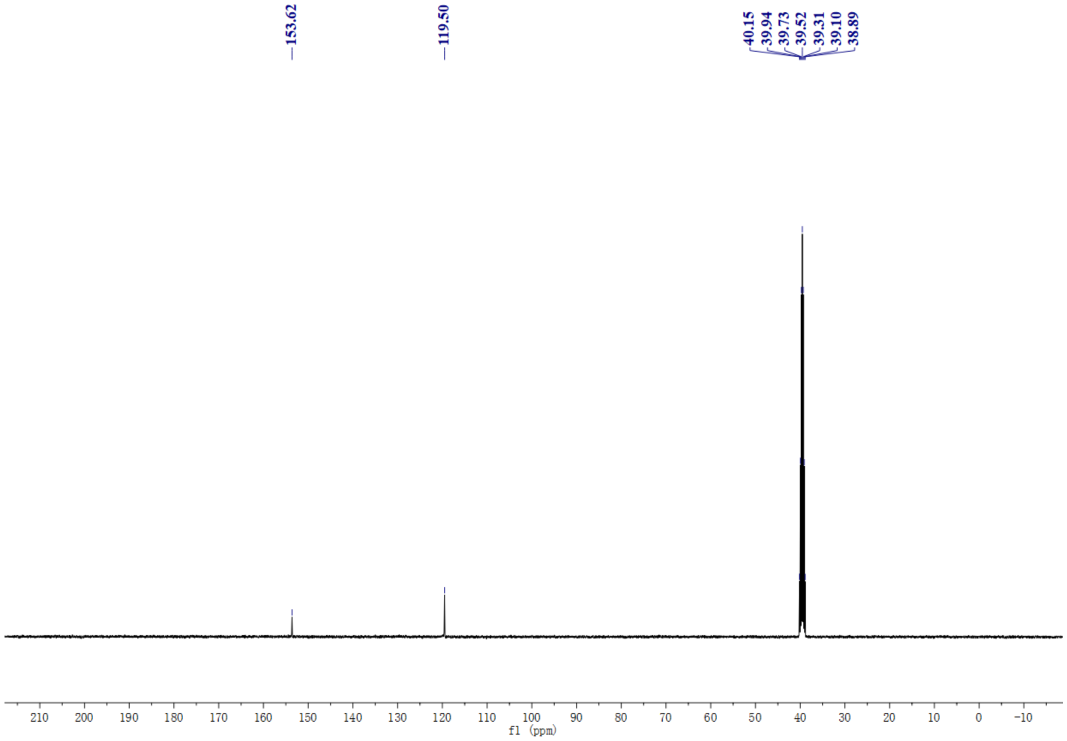

Figure S9. ^{13}C NMR in DMSO-d_6 for *E*-H_2bte.

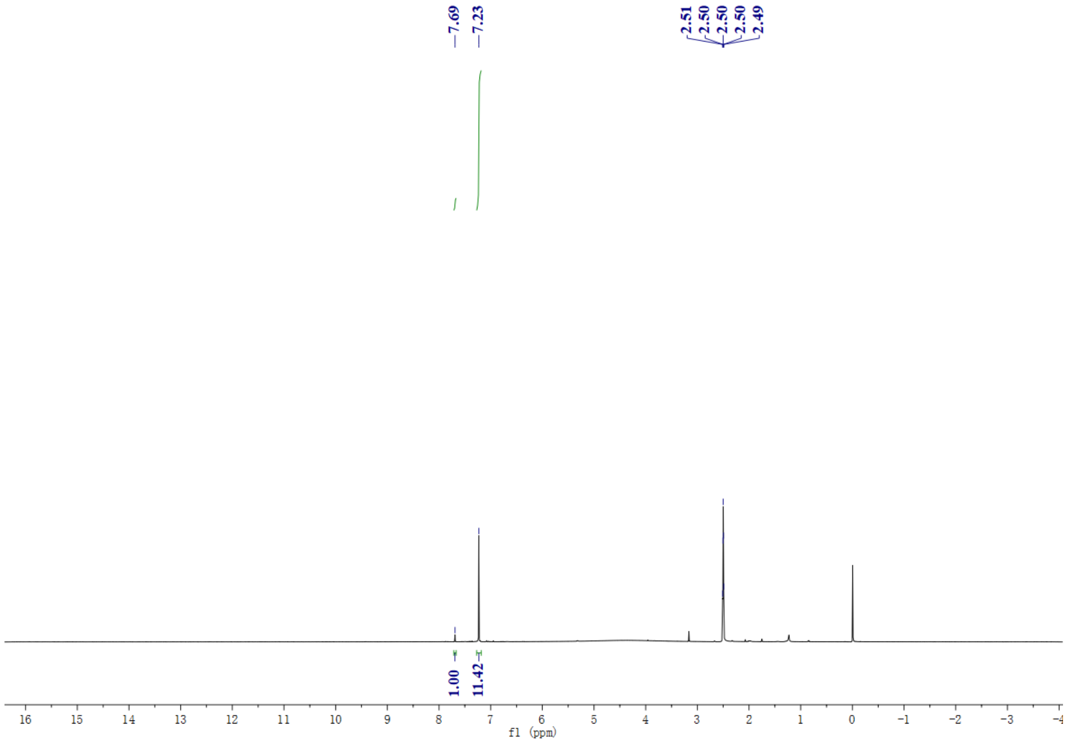

Figure S10. ^{1}H NMR in DMSO-d_6 for *Z*-H_2bte.

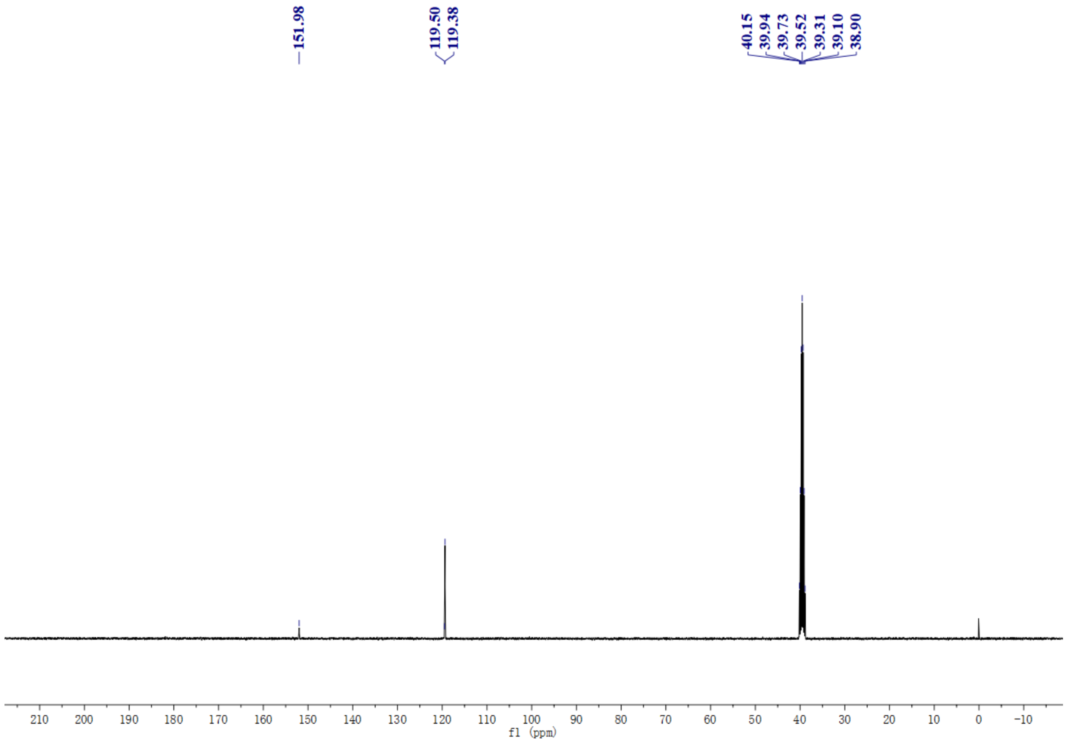

Figure S11. ^{13}C NMR in DMSO-d_6 for *Z*-H_2bte.

a. The NMR spectra of selection of wavelength

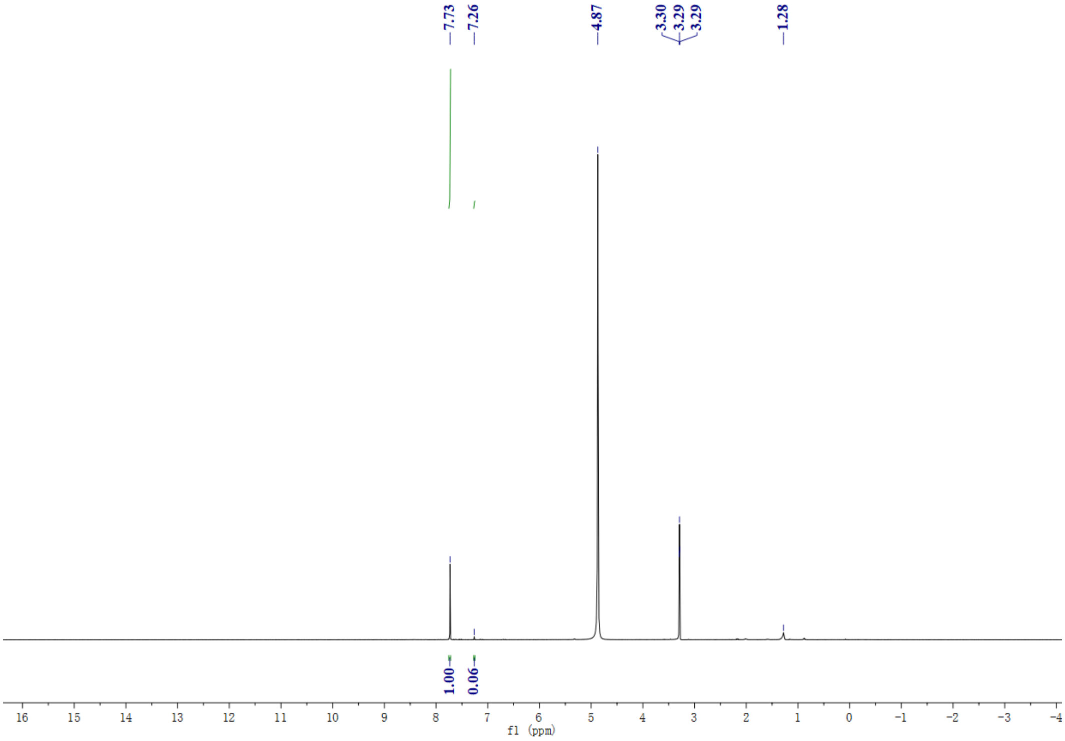

Figure S12. ^{1}H NMR in CD_3OD for *E*-H_2bte irradiated at 260 nm.

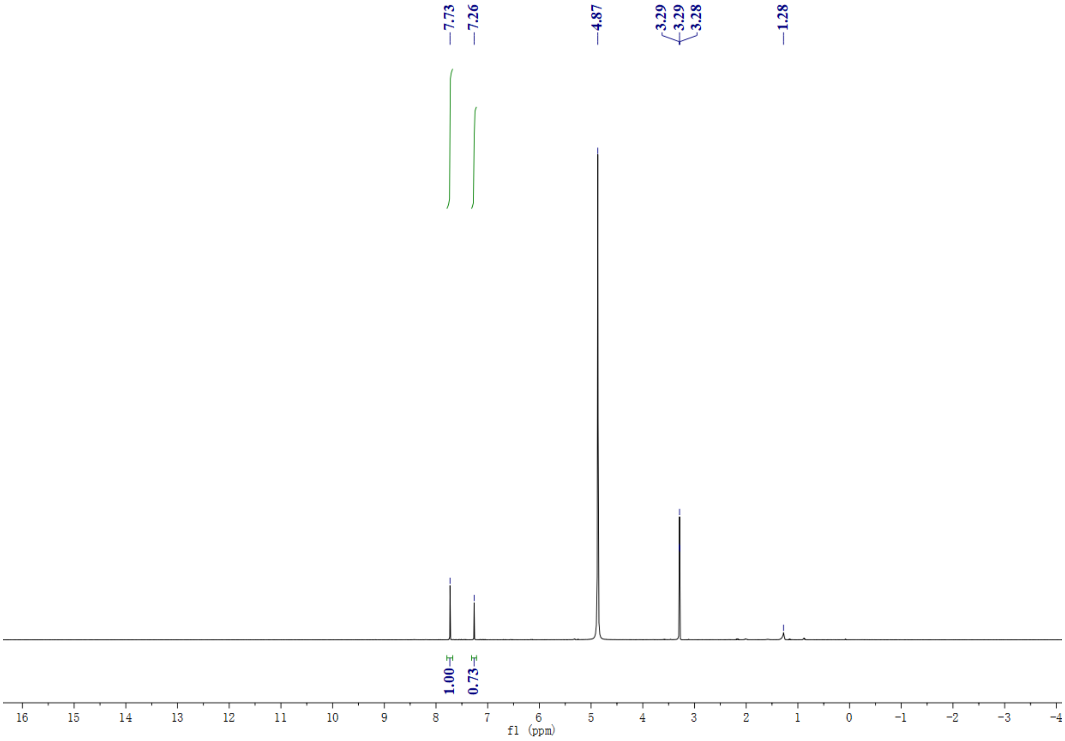

Figure S13. ^{1}H NMR in CD_3OD for *E*-H_2bte irradiated at 300 nm.

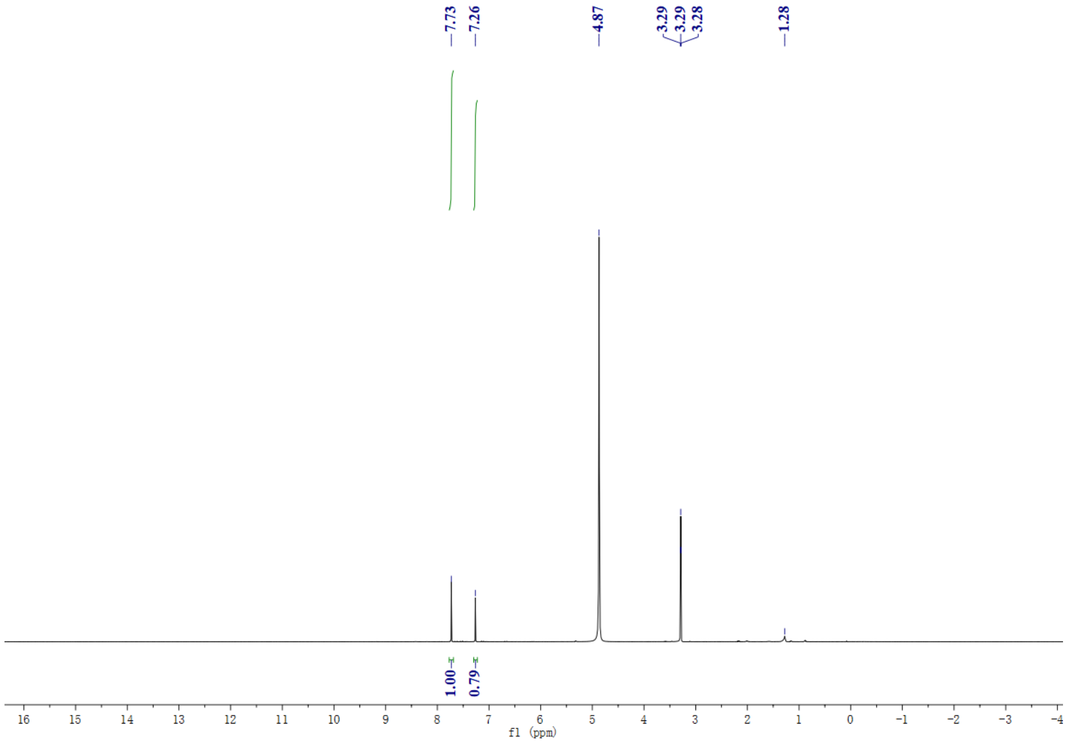

Figure S14. ^{1}H NMR in CD_3OD for *E*-H_2bte irradiated at 340 nm.

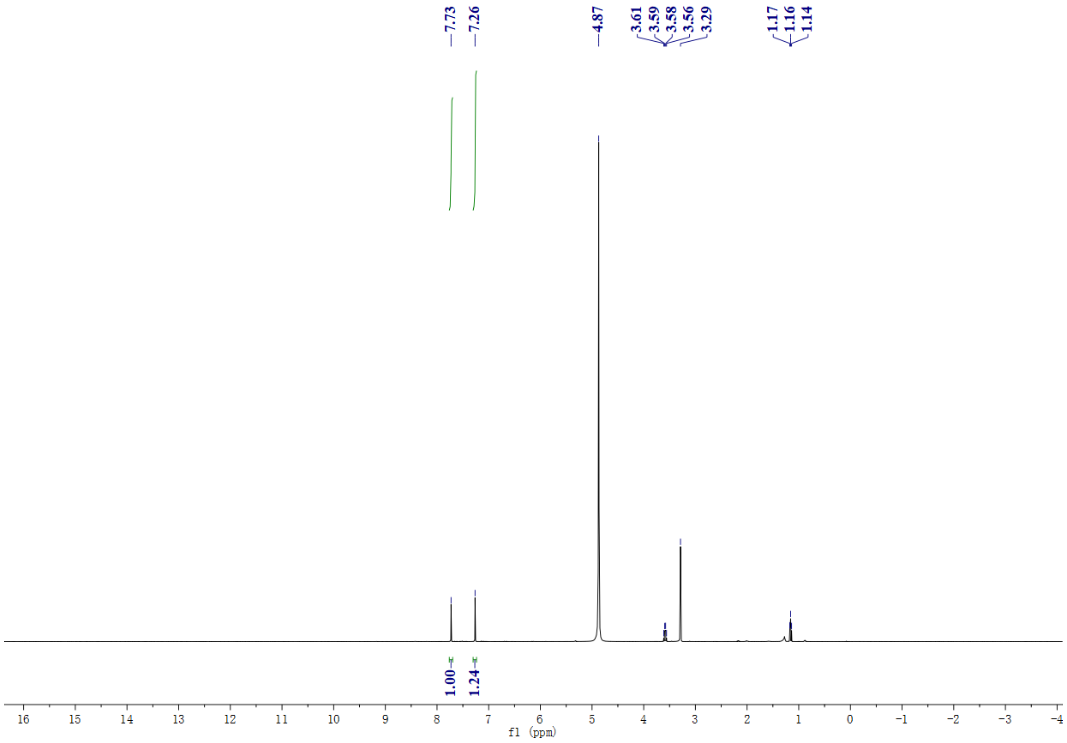

Figure S15. ^{1}H NMR in CD_3OD for *E*-H_2bte irradiated at 365 nm.

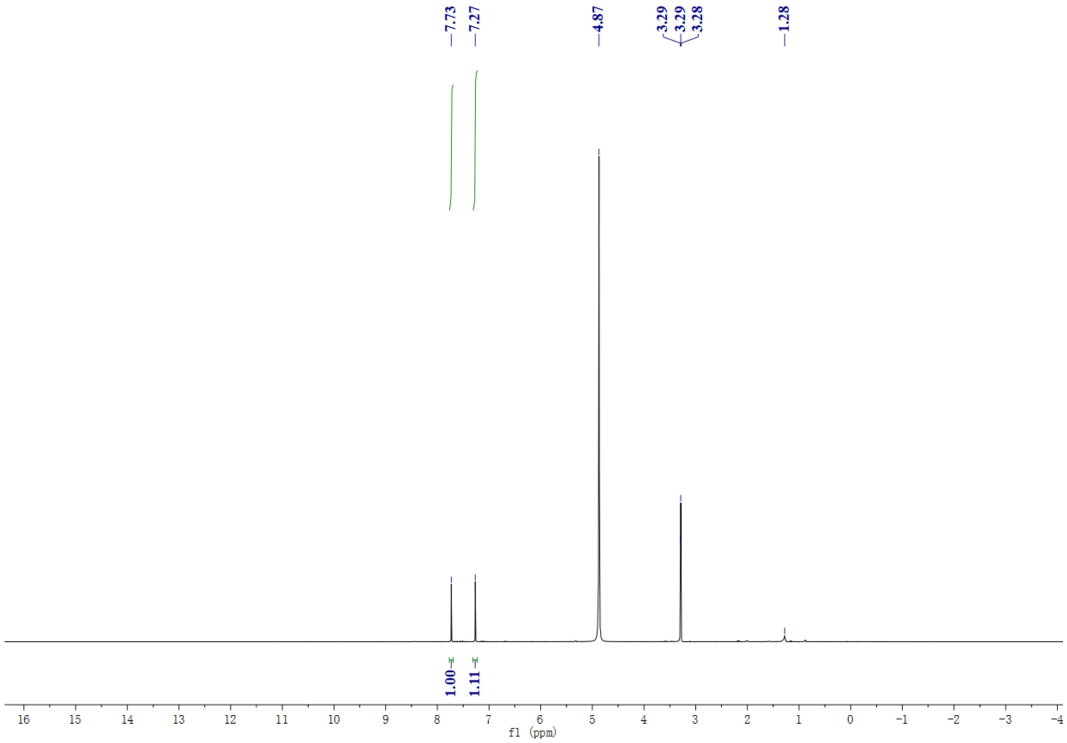

Figure S16. ^{1}H NMR in CD_3OD for *E*-H_2bte irradiated at 395 nm.

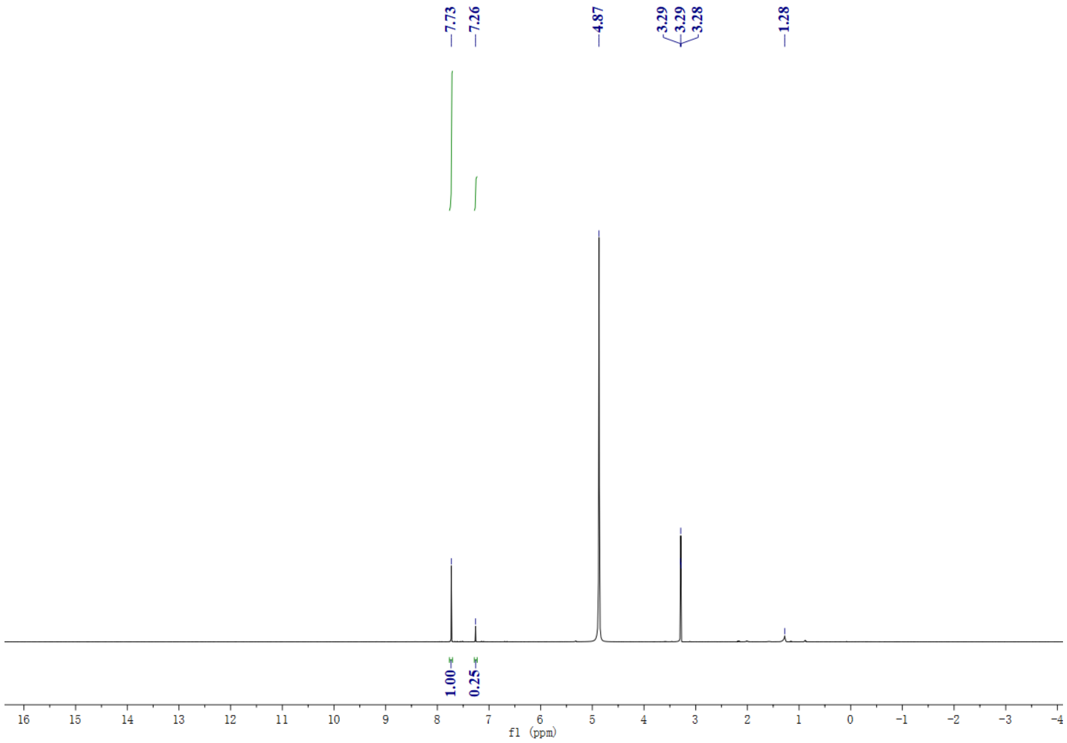

Figure S17. ^{1}H NMR in CD_3OD for *E*-H_2bte irradiated at 440 nm.

b. The NMR spectra of selection of reaction time.

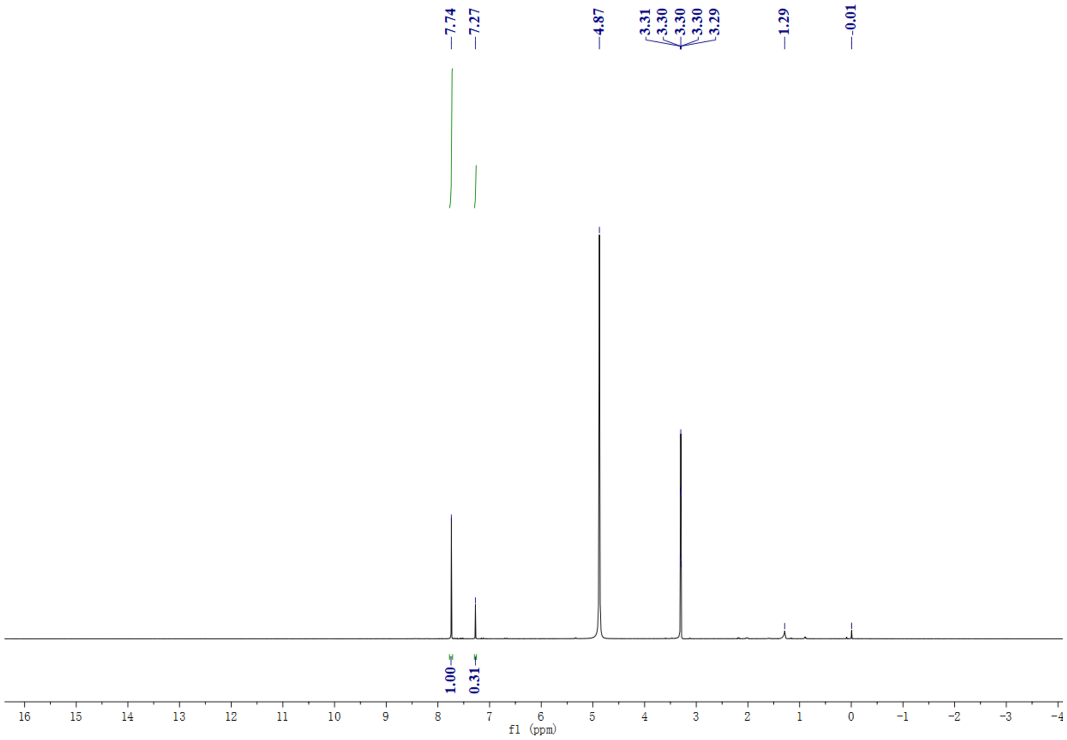

Figure S18. ^{1}H NMR in CD_3OD for *E*-H_2bte irradiated for 5 minutes.

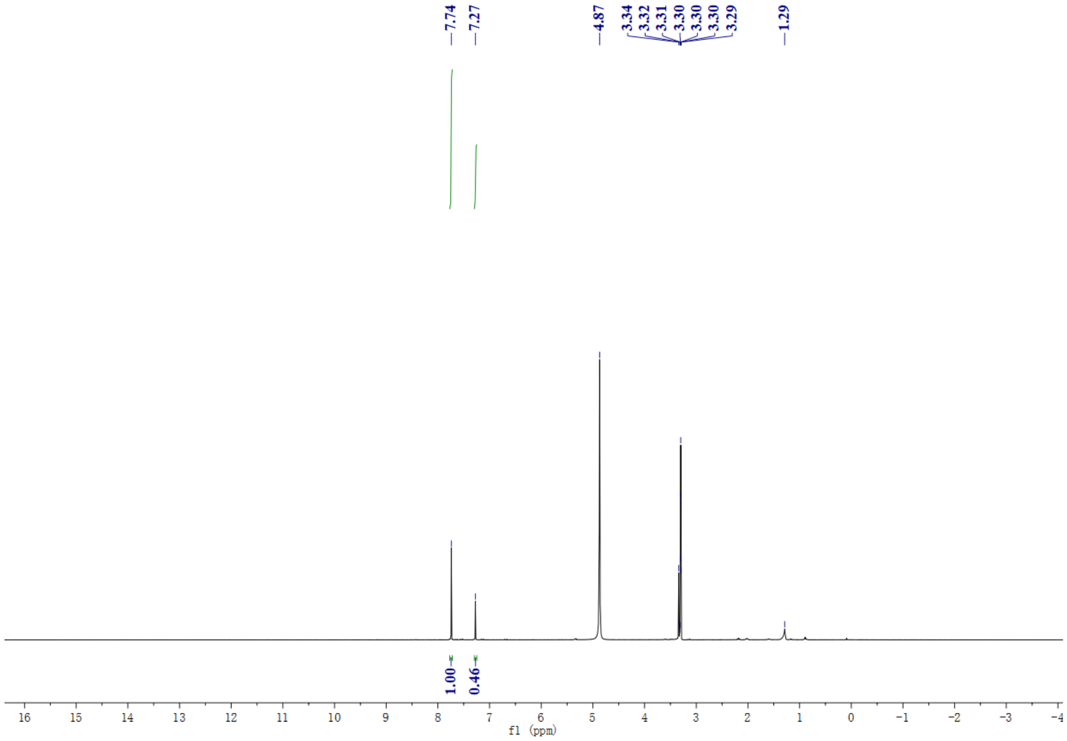

Figure S19. ^{1}H NMR in CD_3OD for *E*-H_2bte irradiated for 10 minutes.

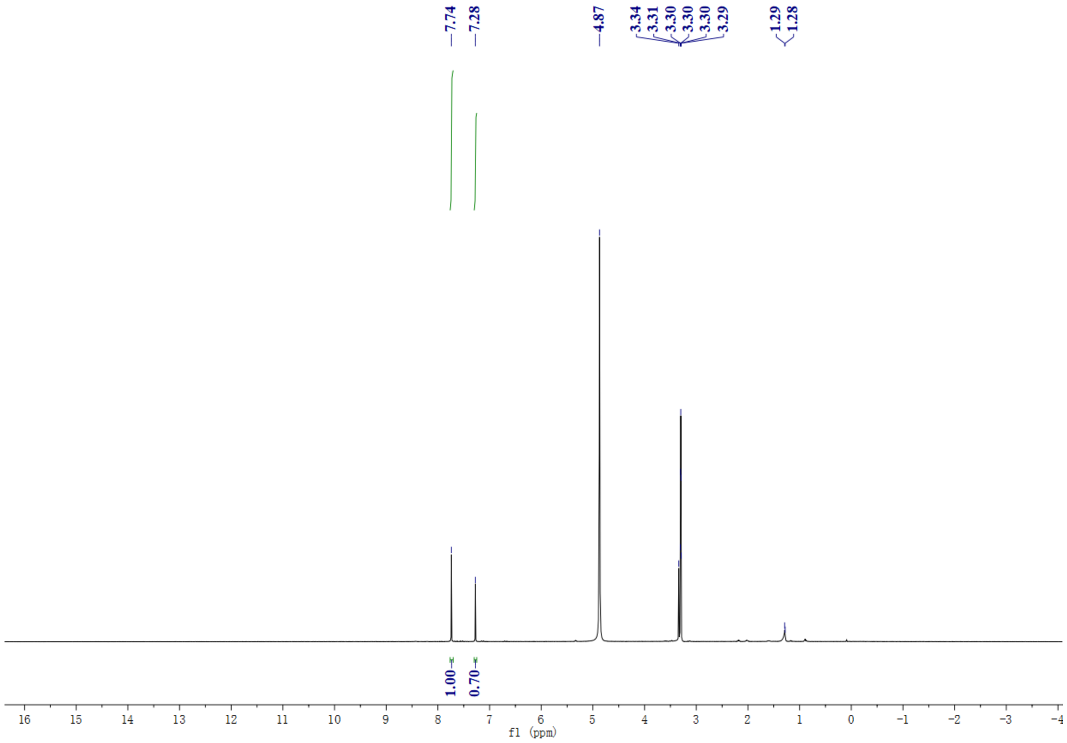

Figure S20. ^{1}H NMR in CD_3OD for *E*-H_2bte irradiated for 20 minutes.

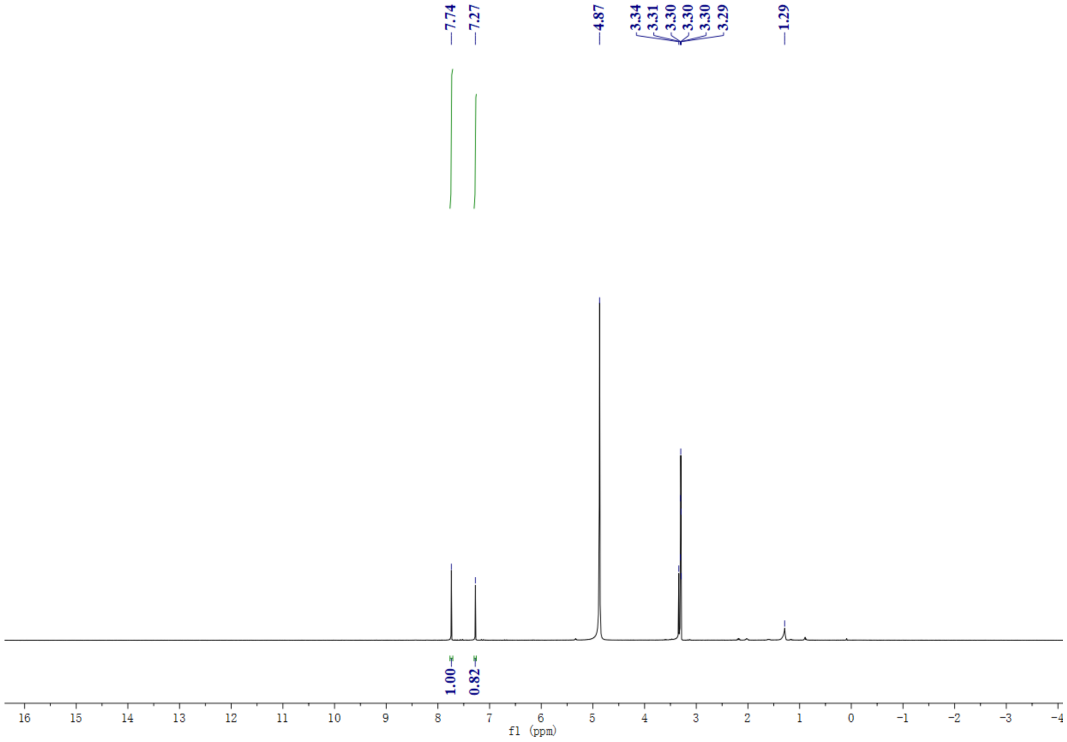

Figure S21. ^{1}H NMR in CD_3OD for *E*-H_2bte irradiated for 30 minutes.

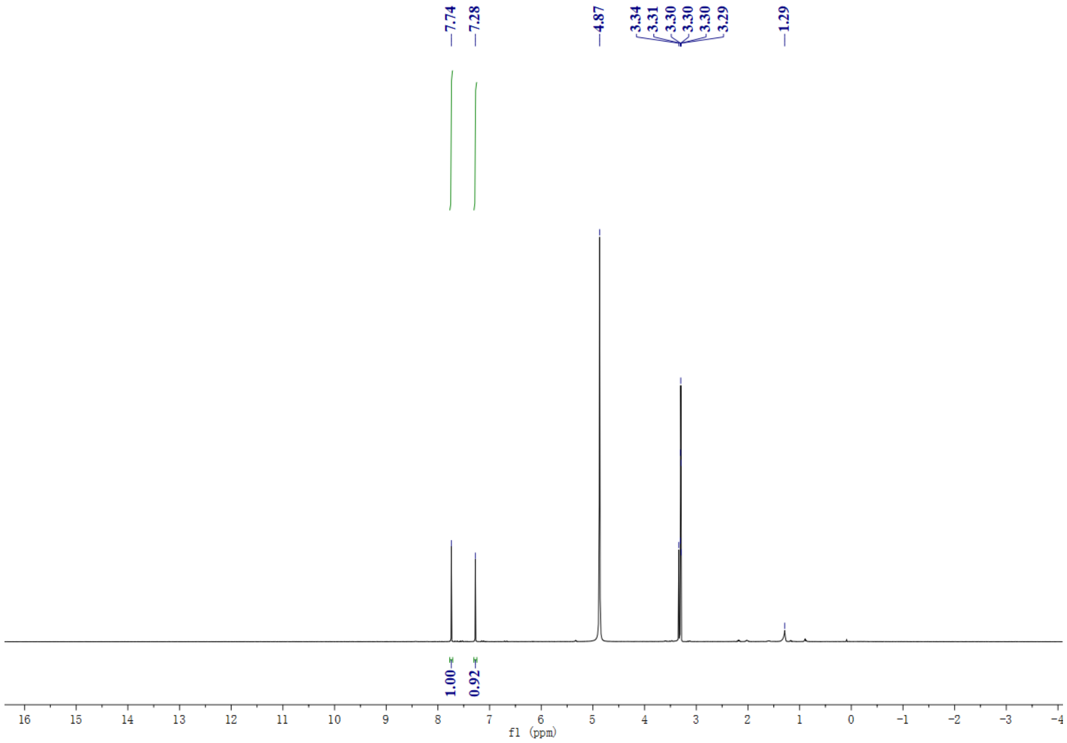

Figure S22. ^{1}H NMR in CD_3OD for *E*-H_2bte irradiated for 40 minutes.

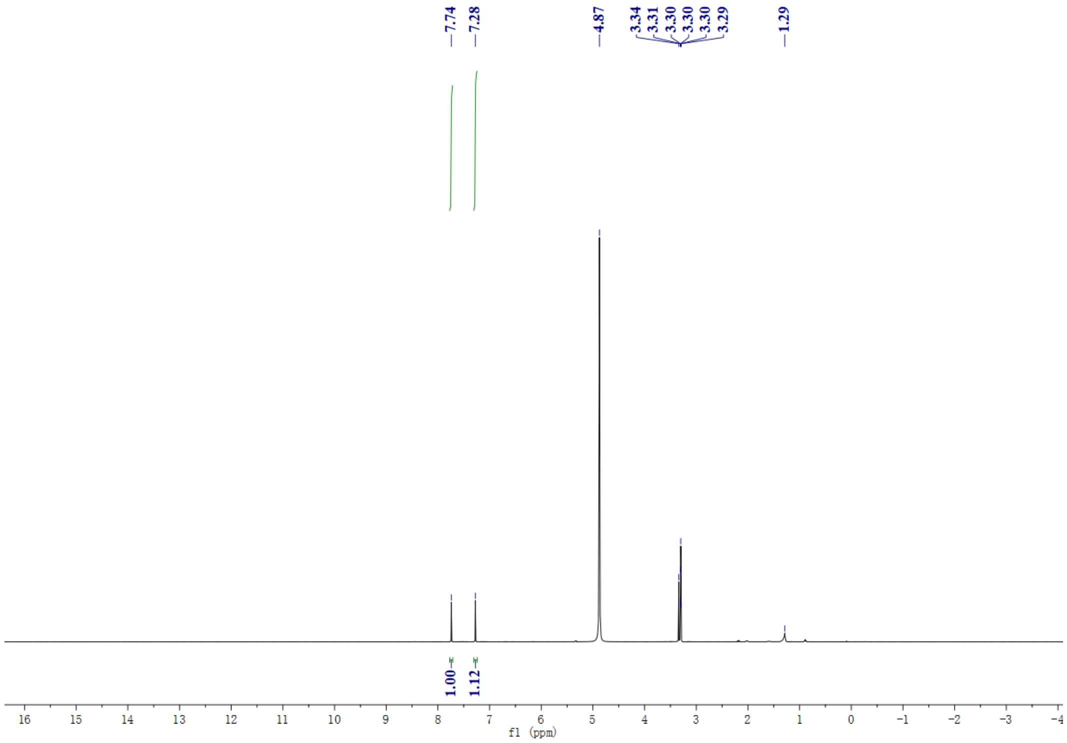

Figure S23. ^{1}H NMR in CD_3OD for *E*-H_2bte irradiated for 50 minutes.

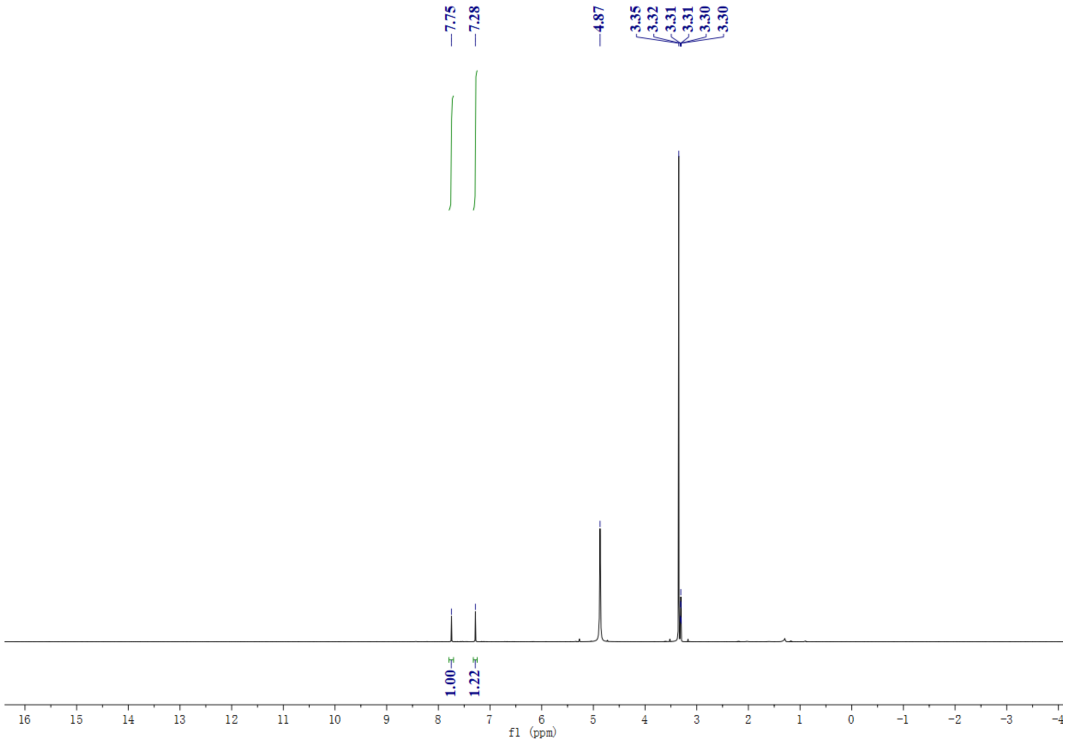

Figure S24. ^{1}H NMR in CD_3OD for *E*-H_2bte irradiated for 60 minutes.

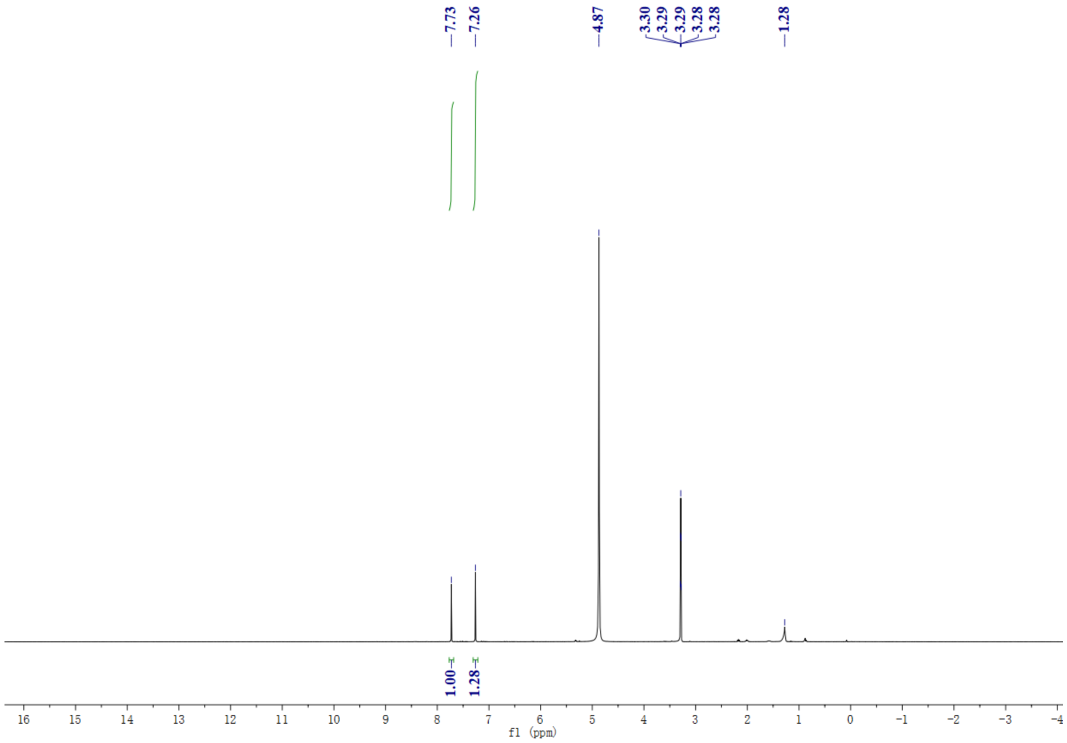

Figure S25. ^{1}H NMR in CD_3OD for *E*-H_2bte irradiated for 80 minutes.

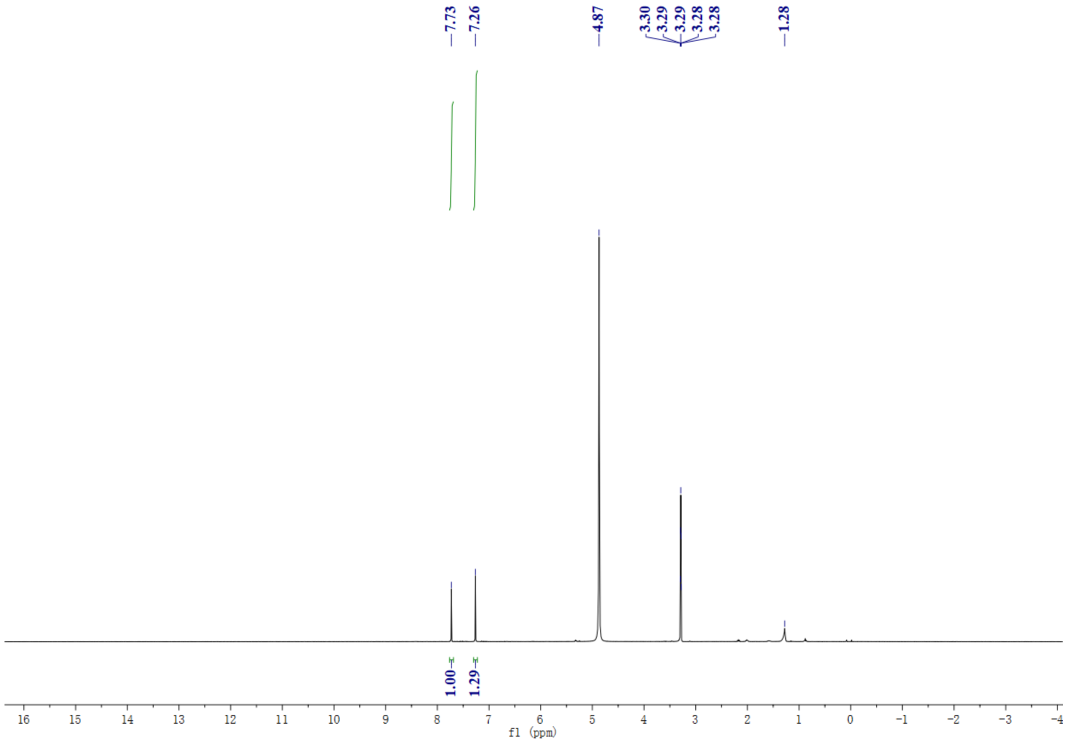

Figure S26. ^{1}H NMR in CD_3OD for *E*-H_2bte irradiated for 100 minutes.

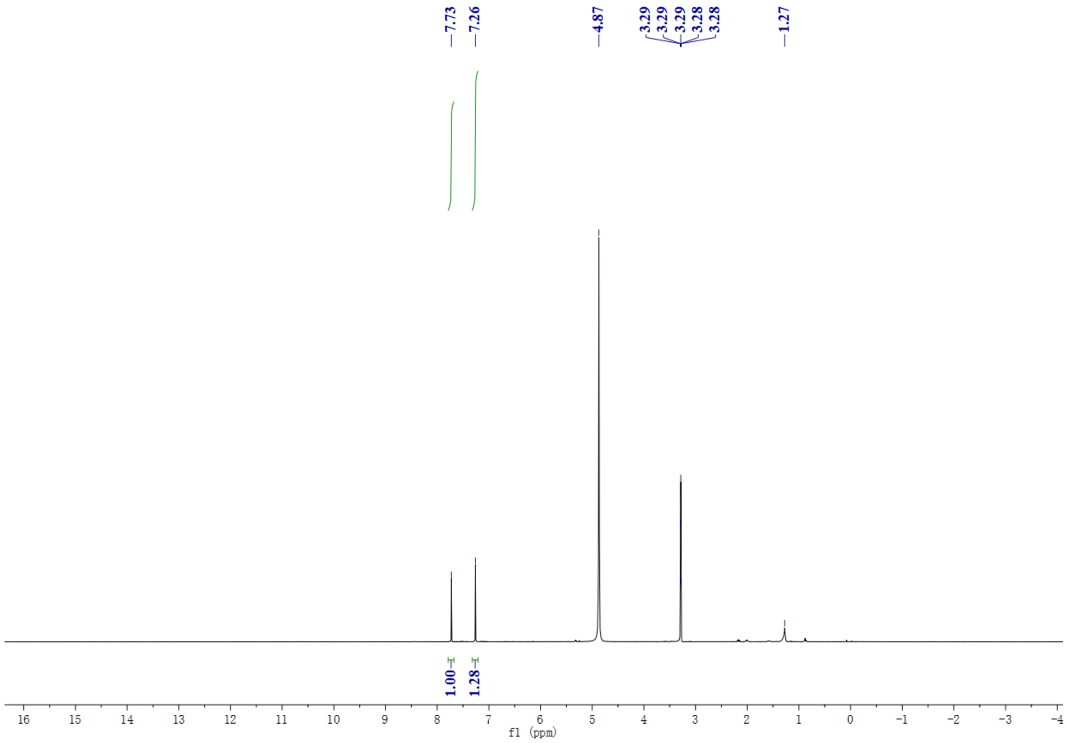

Figure S27. ^{1}H NMR in CD_3OD for *E*-H_2bte irradiated for 120 minutes.

c. The NMR spectra of selection of reaction temperature.

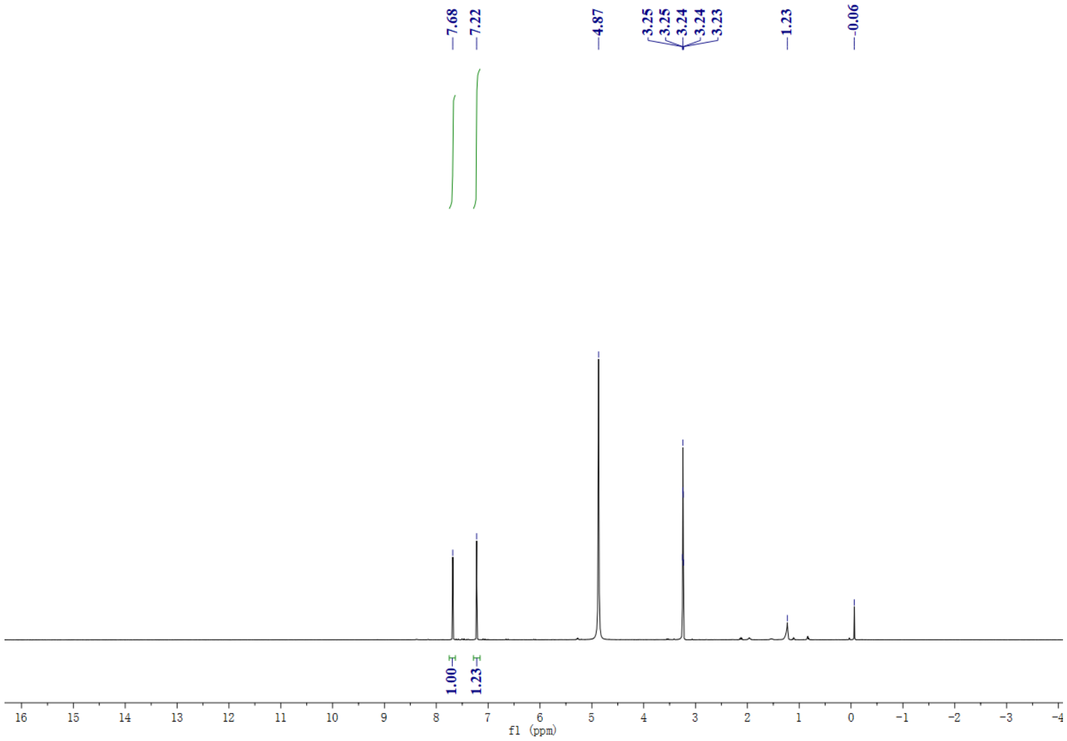

Figure S28. ^{1}H NMR in CD_3OD for *E*-H_2bte irradiated at 0 °C.

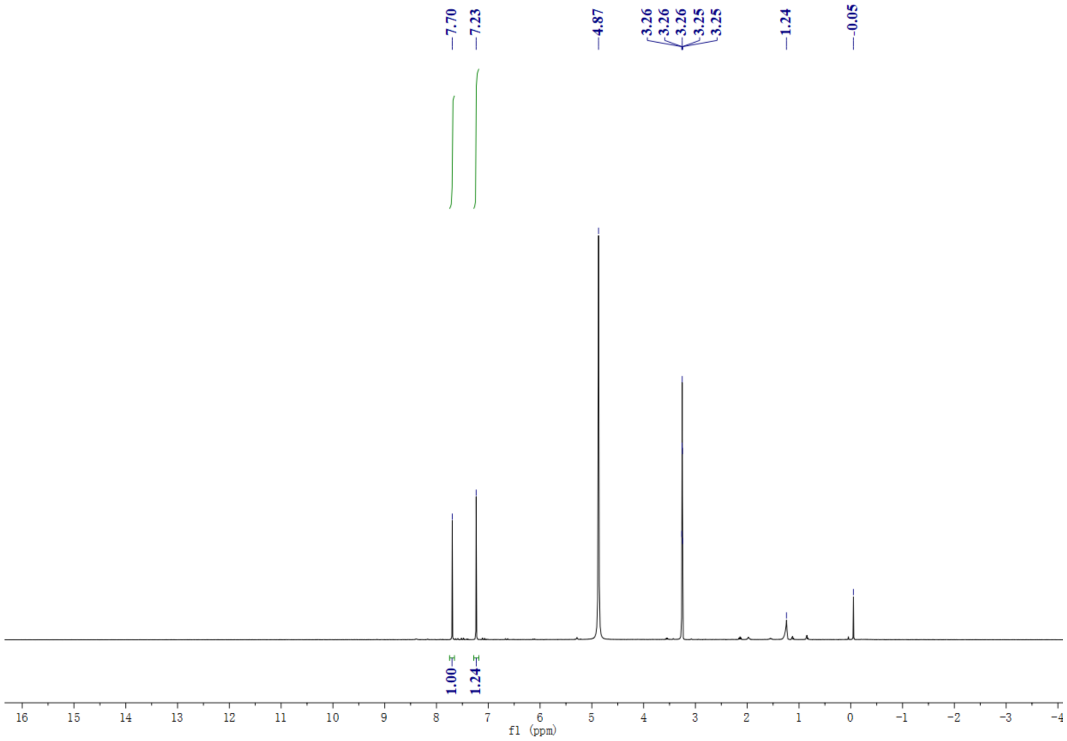

Figure S29. ^{1}H NMR in CD_3OD for *E*-H_2bte irradiated at -20 °C.

d. The NMR spectra of selection of the solvent.

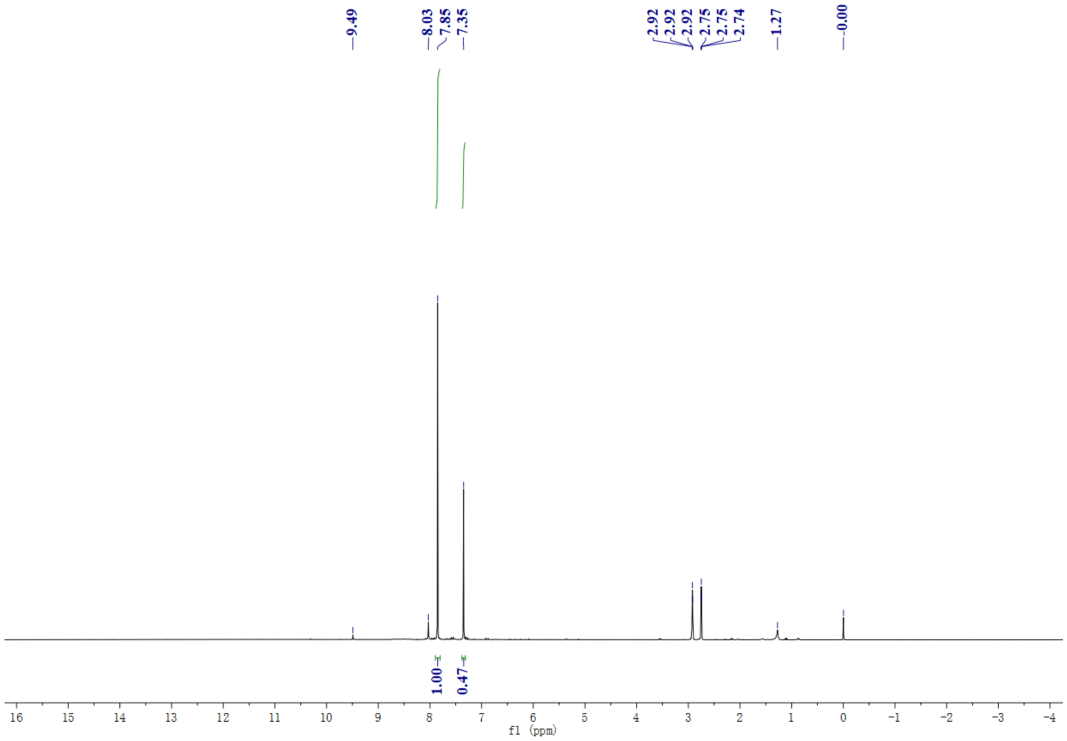

Figure S30. ^{1}H NMR in *N*,*N*-Dimethylformamide-d_7 for *E*-H_2bte irradiated.

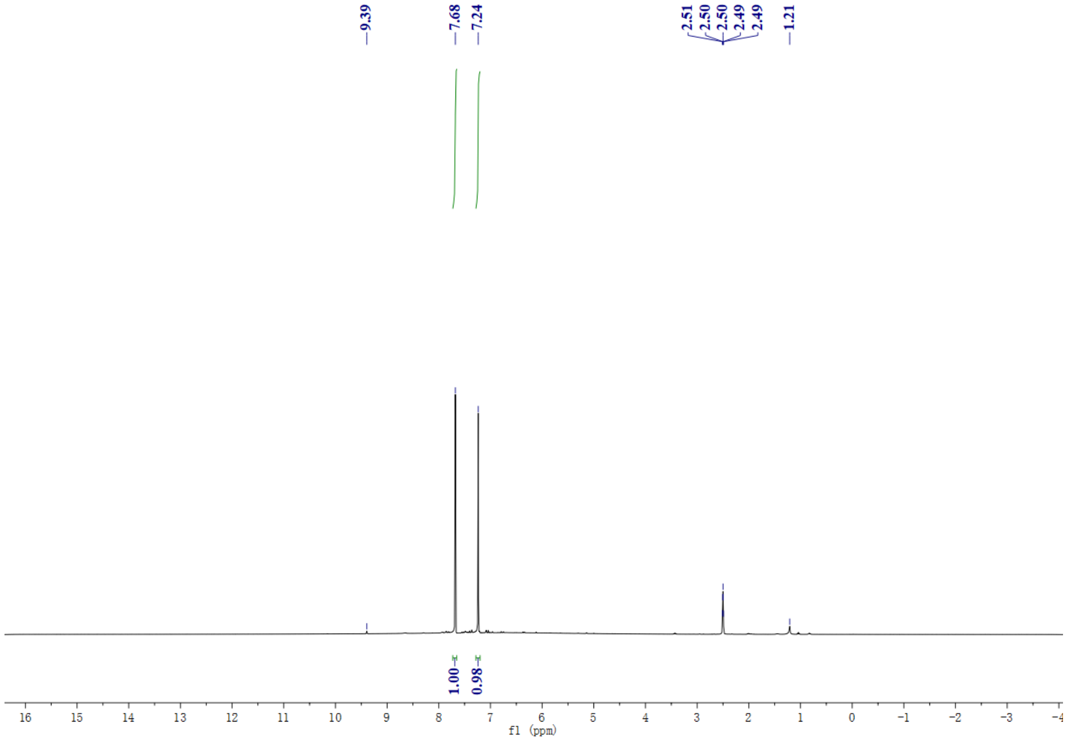

Figure S31. ^{1}H NMR in Dimethyl sulfoxide-d_6 for *E*-H_2bte irradiated.

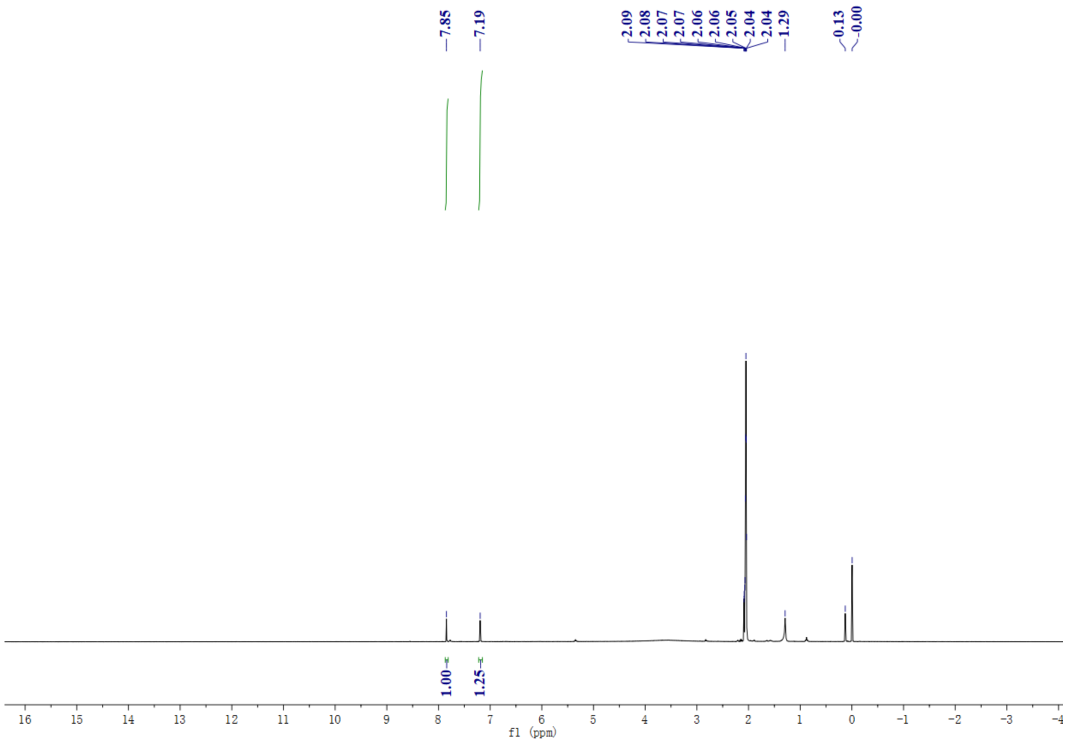

Figure S32. ^{1}H NMR in Acetone-d_6 for *E*-H_2bte irradiated.

e. The NMR spectra of PSS after heating at 100 °C.

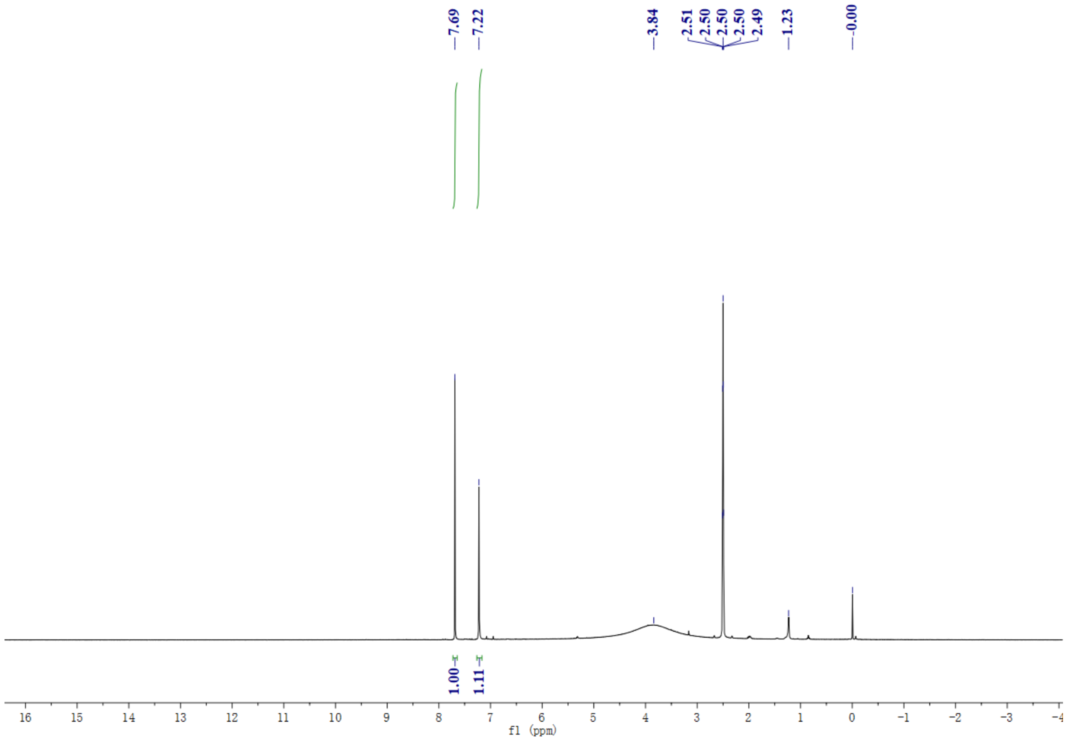

Figure S33. ^{1}H NMR in DMSO-d_6 for PSS.

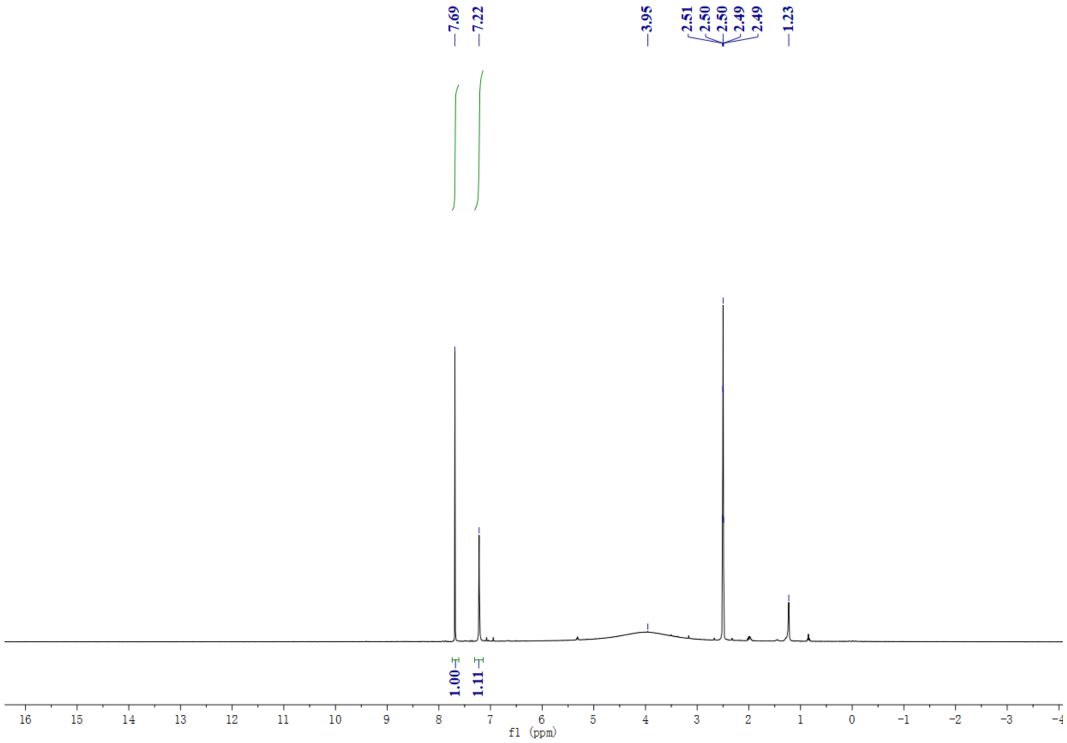

Figure S34. ^{1}H NMR in DMSO-d_6 for PSS after heating at 100 °C.

f. The NMR spectra of Comp. 1 to 4.

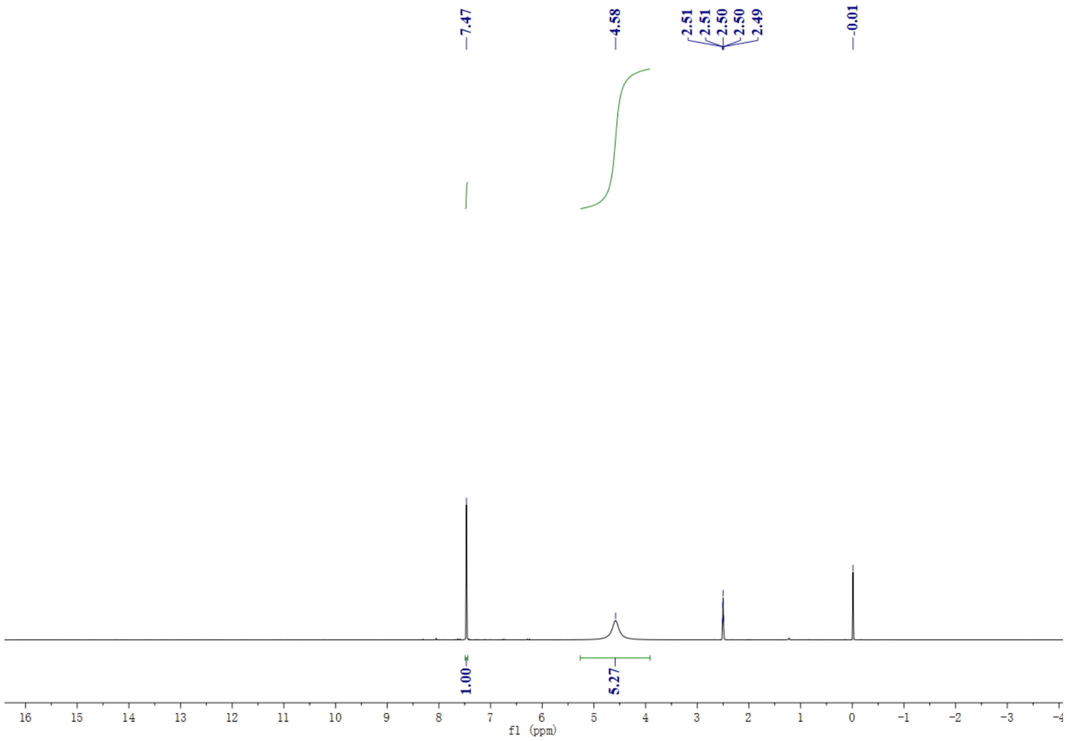

Figure S35. ^{1}H NMR in DMSO-d_6 for Comp.1.

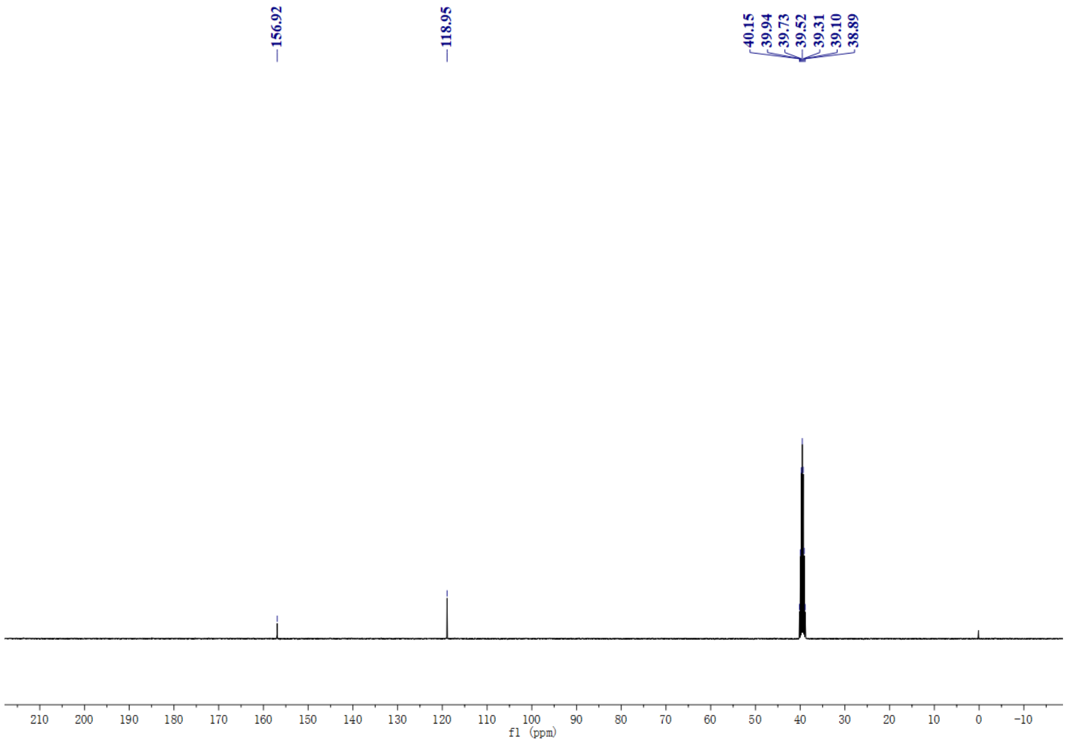

Figure S36. ^{13}C NMR in DMSO-d_6 for Comp.1.

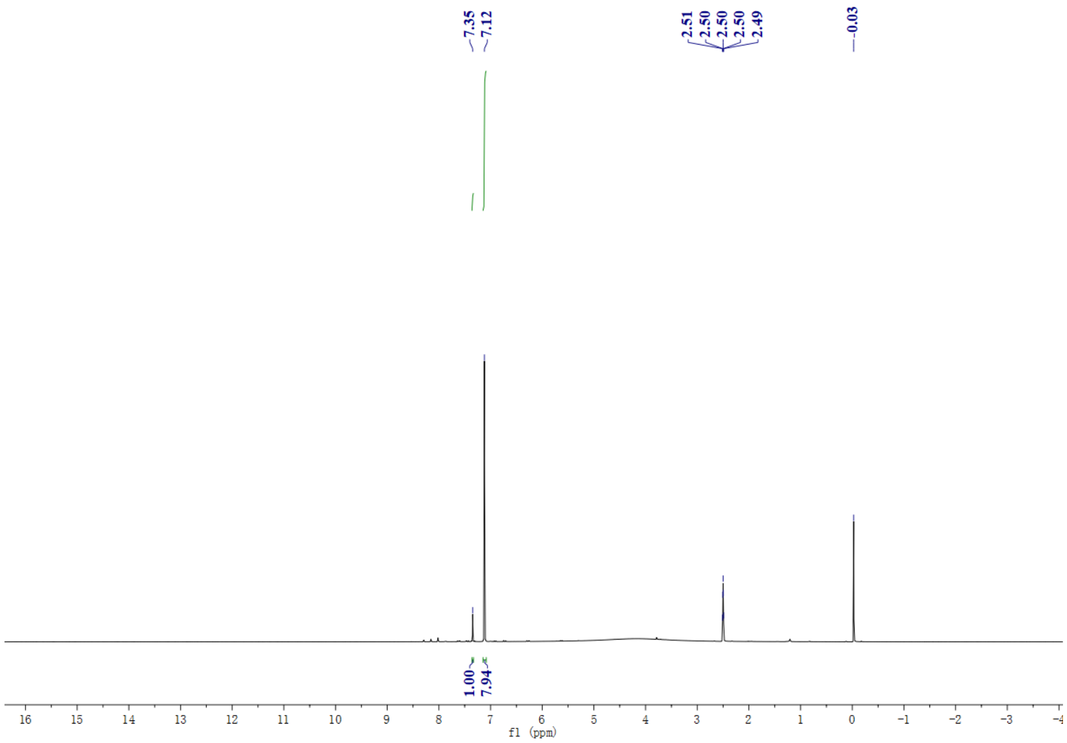

Figure S37. ^{1}H NMR in DMSO-d_6 for Comp.2.

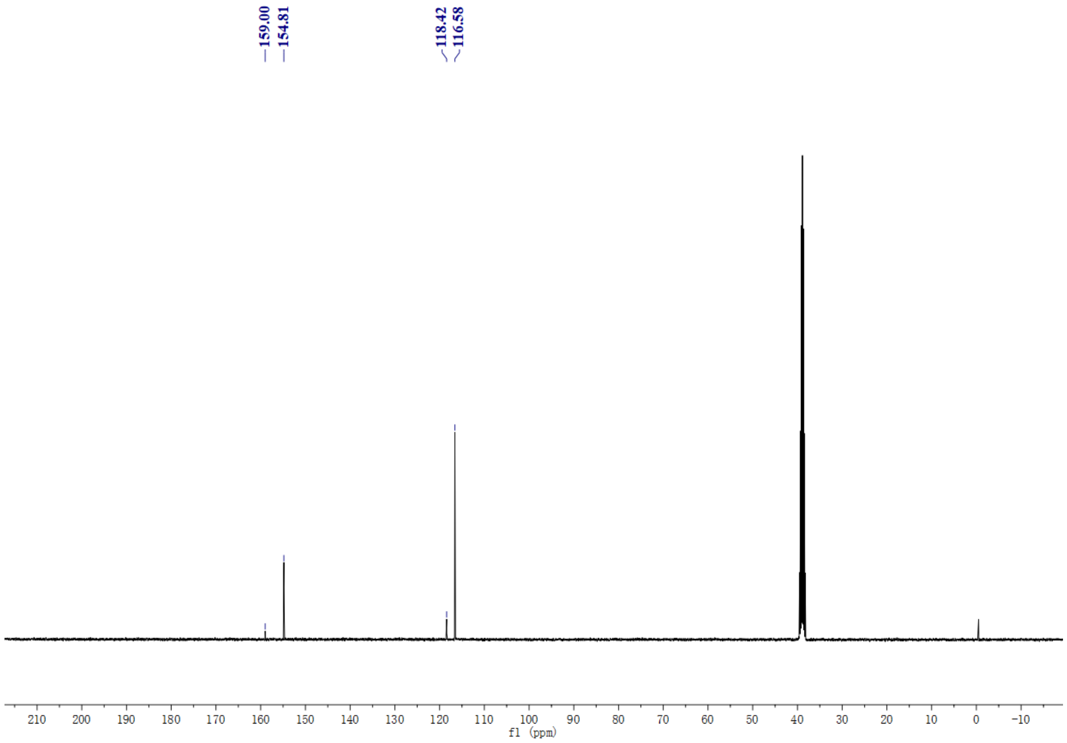

Figure S38. ^{13}C NMR in DMSO-d_6 for Comp.2.

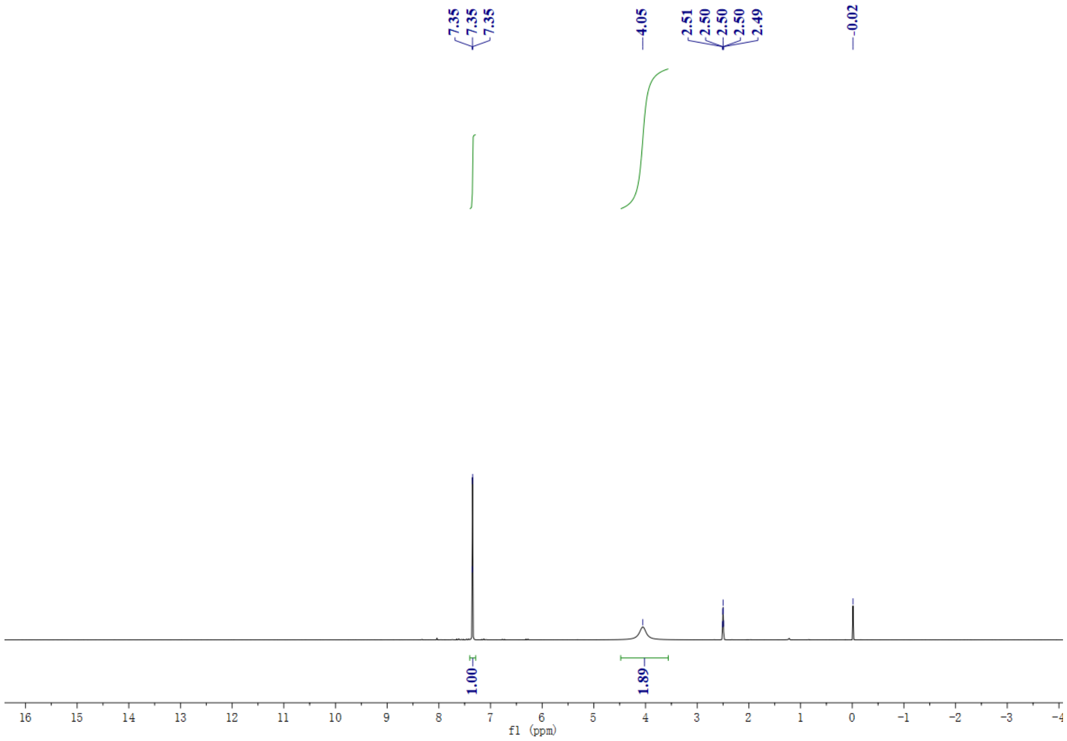

Figure S39. ^{1}H NMR in DMSO-d_6 for Comp.3.

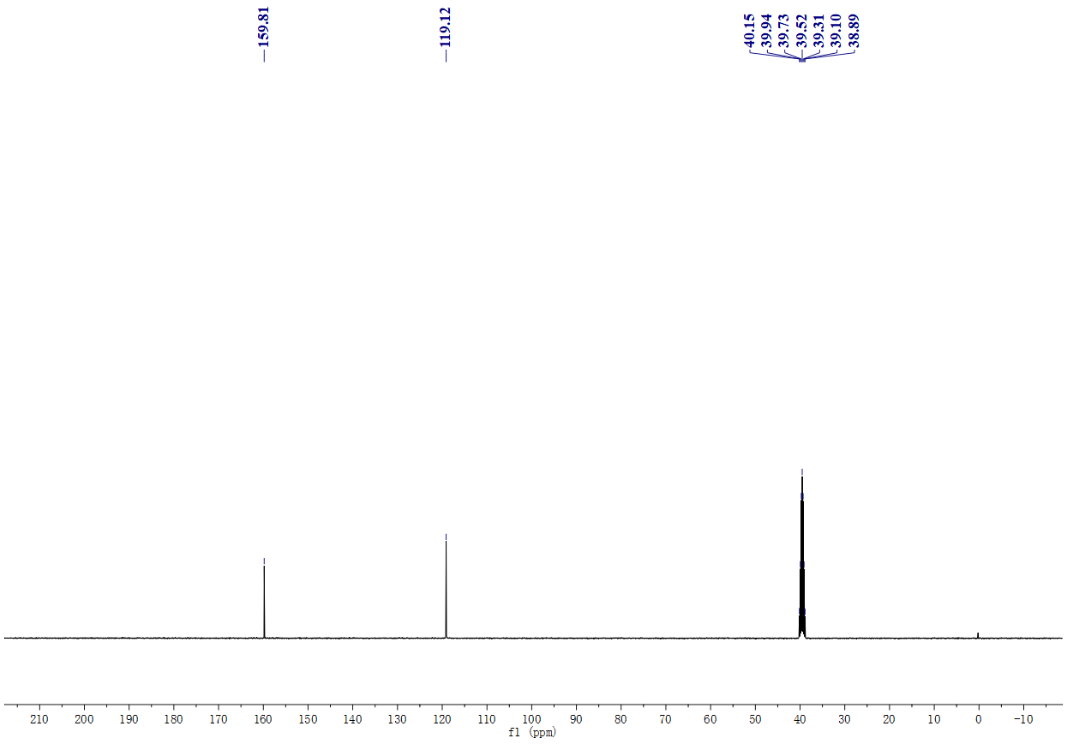

Figure S40. ^{13}C NMR in DMSO-d_6 for Comp.3.

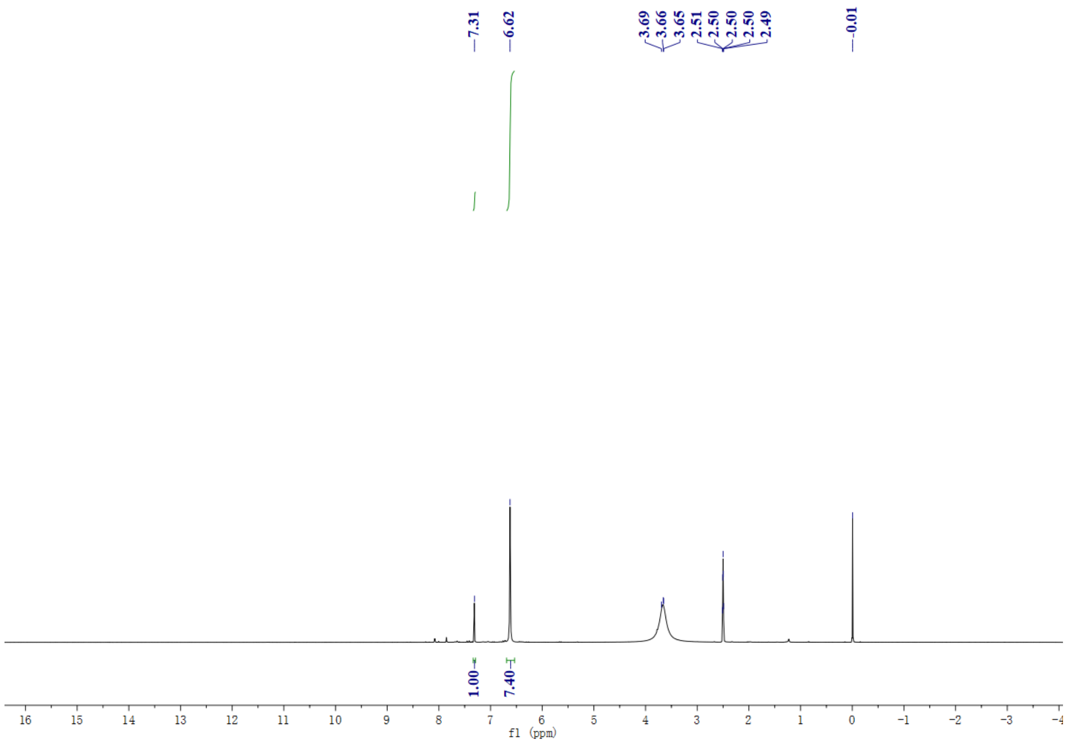

Figure S41. ^{1}H NMR in DMSO-d_6 for Comp.4.

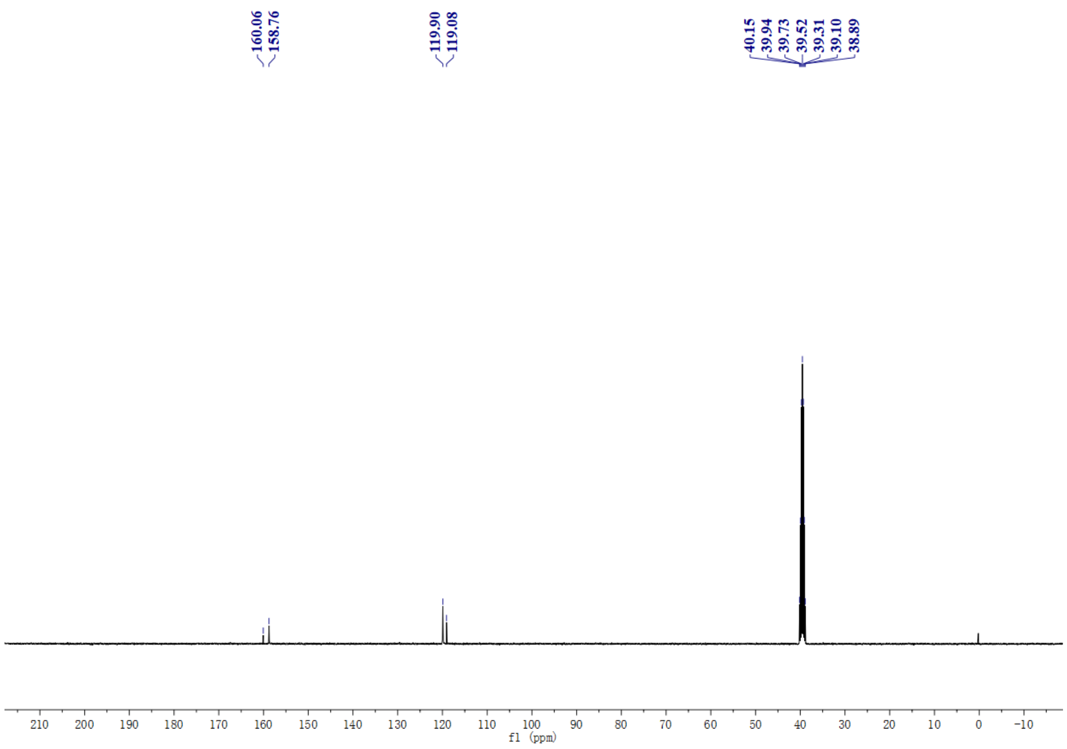

Figure S42. ^{13}C NMR in DMSO-d_6 for Comp.4.

5. Powder X-ray diffraction patterns

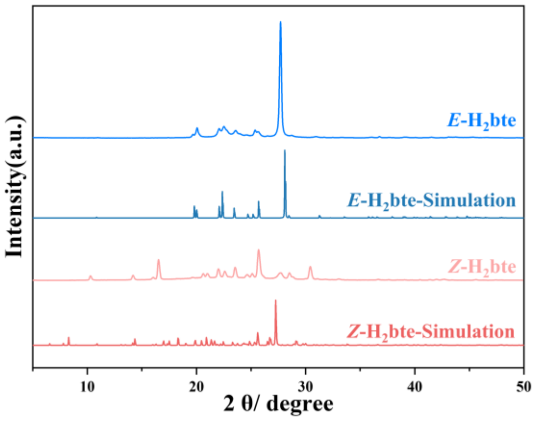

Figure S43. The powder X-ray diffraction patterns of *E*-H_2bte and *Z*-H_2bte.

6. Infrared spectrum

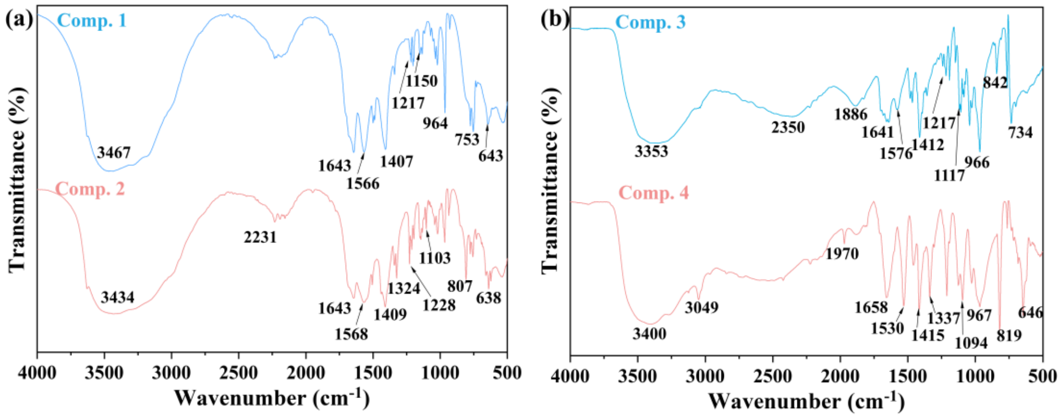

Figure S44. The infrared spectrum of Comp. 1–Comp. 4.

7. TG/DSC curve

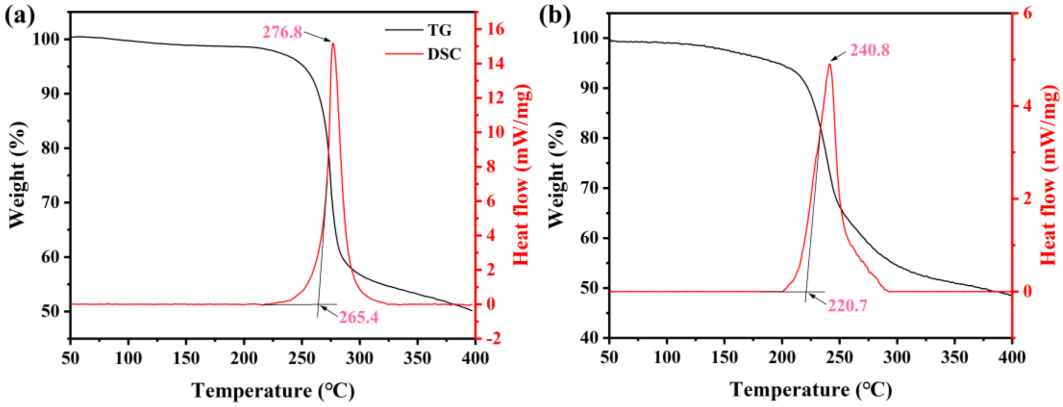

Figure S45. (a) The TG/DSC curve of *E*-H_2bte. (b) The TG/DSC curve of *Z*-H_2bte.

7. References

[S1] T. Lu, F. Chen, Multiwfn: a multifunctional wavefunction analyzer, J. Comput. Chem., 2012, 33(5), 580–592.

[S2] T. Lu, F. Chen, Quantitative analysis of molecular surface based on improved marching tetrahedra algorithm, J. Mol. Graph. Model., 2012, 38, 314–323.

[S3] E. R. Johnson, S. Keinan, A. J. Cohen, W. Yang, Revealing noncovalent interactions, J. Am. Chem. Soc., 2010, 132(18), 6498–6506.

[S4] W. Humphrey, A. Dalk, K. Schulten, VMD: Visual molecular dynamics, J. Mol. Graphics., 1996, 14(1), 33–38.

[S5] M. S. Westwell, M. S. Searle, D. J. Wales, D. H. Wiliams, Empirical Correlations between Thermodynamic Properties and Intermolecular Forces, J. Am. Chem. Soc., 1995, 117, 5013-5015.
